# Identification of Natural Isonitriles Through Ligation to an Azomethine Imine Probe

**DOI:** 10.1002/chem.202503642

**Published:** 2026-01-15

**Authors:** Maurice P. Biedermann, Alexander Brachmann, Shurui Mai, Athanasios Markos, Markus Künzler, Jörn Piel, Helma Wennemers

**Affiliations:** ^1^ Laboratory of Organic Chemistry D‐CHAB ETH Zürich Zürich Switzerland; ^2^ Institute of Microbiology D‐BIOL ETH Zürich Zürich Switzerland

**Keywords:** azomethine imines, bioorthogonal chemistry, isonitriles, natural products, reactivity‐based screening

## Abstract

Natural isonitriles are promising lead compounds for medicinal chemistry. Their identification is, however, challenging due to the hydrolytic lability of the isonitrile group. Here, we use the azomethine imine (AMI)–isonitrile ligation in a reactivity‐based screening protocol for the chemoselective derivatization of natural isonitriles. The herein developed AMI probe rapidly reacts with isonitriles—primary, secondary, tertiary, as well as aromatic—to stable conjugates. A dibromide mass tag enables the detection of low‐abundance isonitriles, even in complex biological matrices. The ligation establishes a new stereogenic center, thereby allowing facile distinction between achiral and chiral isonitriles by the formation of racemates or diastereoisomers, respectively. In addition, a unique reactivity of isonitriles bearing an α‐COOH group was unraveled. This AMI probe enabled the detection of known bacterially produced isonitriles and the identification of a novel fungal isonitrile.

## Graphical abstract

The azomethine imine (AMI)–isonitrile (NC) ligation allowed for the chemoselective detection of natural isonitriles in bacteria and fungi. The reactivity‐based screening establishes a new stereogenic center, thereby allowing for facile distinction between chiral and achiral isonitriles. The work also unraveled a unique reactivity of isonitriles bearing an α‐COOH group.

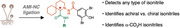

## 1 Introduction

**FIGURE 1:**
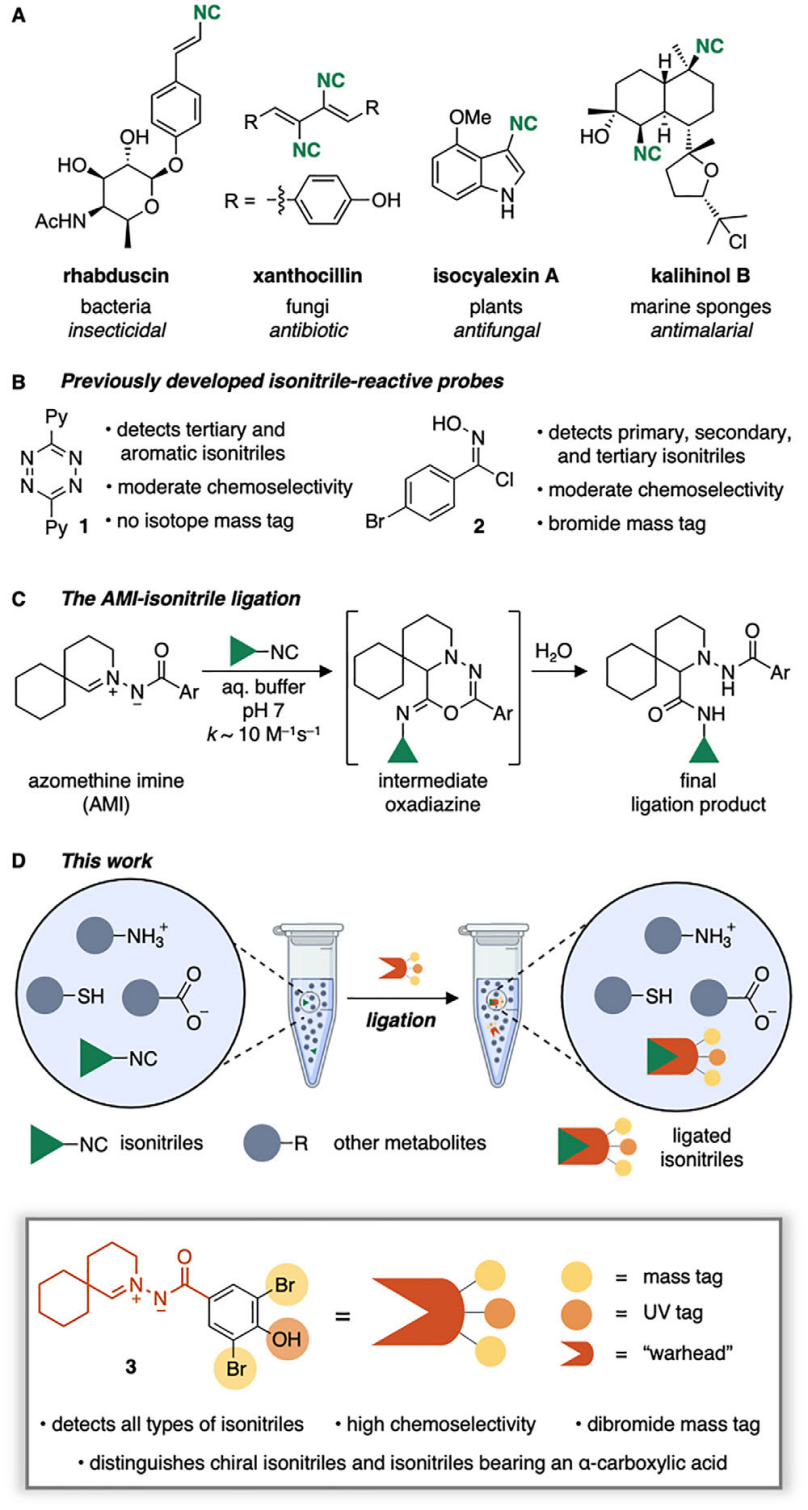
(A) Examples of natural isonitriles, their source, and bioactivity. (B) Previously introduced RBS probes, tetrazine **1** and chlorooxime **2**, for the detection of natural isonitriles. (C) AMI‐isonitrile ligation. (D) Concept for the identification of natural isonitriles with AMI probe **3**.

Isonitrile‐containing natural products have been identified in various organisms, such as bacteria, fungi, plants, and marine sponges (Figure 1A) [1, 2, 3, 4, 5, 6, 7]. Natural isonitriles exhibit potent antibacterial, antifungal and antimalarial activities, making them promising lead compounds for drug development [3, 4, 5, 6, 7, 8]. The identification and isolation of natural isonitriles, however, are often challenging due to the lability of the isonitrile group to acid‐catalyzed hydrolysis [9]. Hydrolysis can even occur during standard high‐performance liquid chromatography high‐resolution mass spectrometry (HPLC‐HRMS) analysis [10] or reverse‐phase (RP)‐HPLC purification [11]. Effective trapping of the isonitrile prior to analysis is therefore crucial for capturing and utilizing the breadth of isonitriles offered by nature. An ideal trapping probe reacts chemoselectively with the isonitrile, forms a stable product, and features a moiety that facilitates detection of the product, for example, through a characteristic isotope mass tag, a lipophilic UV‐Vis chromophore, or an ionizable group. Initial approaches for such a reactivity‐based screening (RBS) employed 3,6‐dipyridyltetrazine **1** [12, 13]. This probe forms stable products with tertiary and aromatic isonitriles, but the products of primary or secondary isonitriles undergo spontaneous hydrolysis (Figure 1B, left) [12, 13, 14, 15, 16]. Furthermore, probe **1** exhibits significant cross‐reactivity with naturally occurring alkenes [12, 17] and degrades under aqueous conditions [18], further limiting its use as a chemoselective probe.

We introduced chlorooxime‐based probe **2**, which forms stable ligation products with primary, secondary, and tertiary aliphatic isonitriles (Figure 1B, right) [19, 20]. The introduction of a bromide mass tag facilitates MS‐based detection of the formed conjugates and enabled the identification of an isonitrile terpene from a marine sponge [19]. Despite these benefits, lability in water over time and cross‐reactivity with thiols limit the value of the chlorooxime probe [20, 21]. For the effective mining of natural isonitriles, a probe that reacts with any type of isonitrile and allows for facile detection and isolation is, therefore, still needed.

Recently, we developed the azomethine imine (AMI)‐isonitrile ligation (Figure 1C) [22, 23]. This reaction is fast at pH 7 (∼10 M^−1^s^−1^) and even faster at acidic pH (∼100 M^−1^s^−1^ at pH 6) and features exquisite chemoselectivity. Importantly, AMIs and their ligation products with isonitriles are stable under aqueous conditions, including in cell lysate [22, 23]. We, therefore, envisioned an appropriately substituted AMI as an ideal isonitrile‐targeting RBS probe. Herein, we show how the AMI‐isonitrile ligation allowed for the detection of the known bacterial natural product rhabduscin and a previously postulated, but so far unidentified, fungal isonitrile from crude biological extracts. Furthermore, we show that the ligation forms, in contrast to previously reported isonitrile‐reactive probes, stable products with primary, secondary, tertiary, and aromatic isonitriles and detects whether the isonitrile‐containing natural product is chiral.

## 2 Results and Discussion

### 2.1 Design and Synthesis of a Universal Isonitrile‐Targeting RBS Probe

**FIGURE 2:**
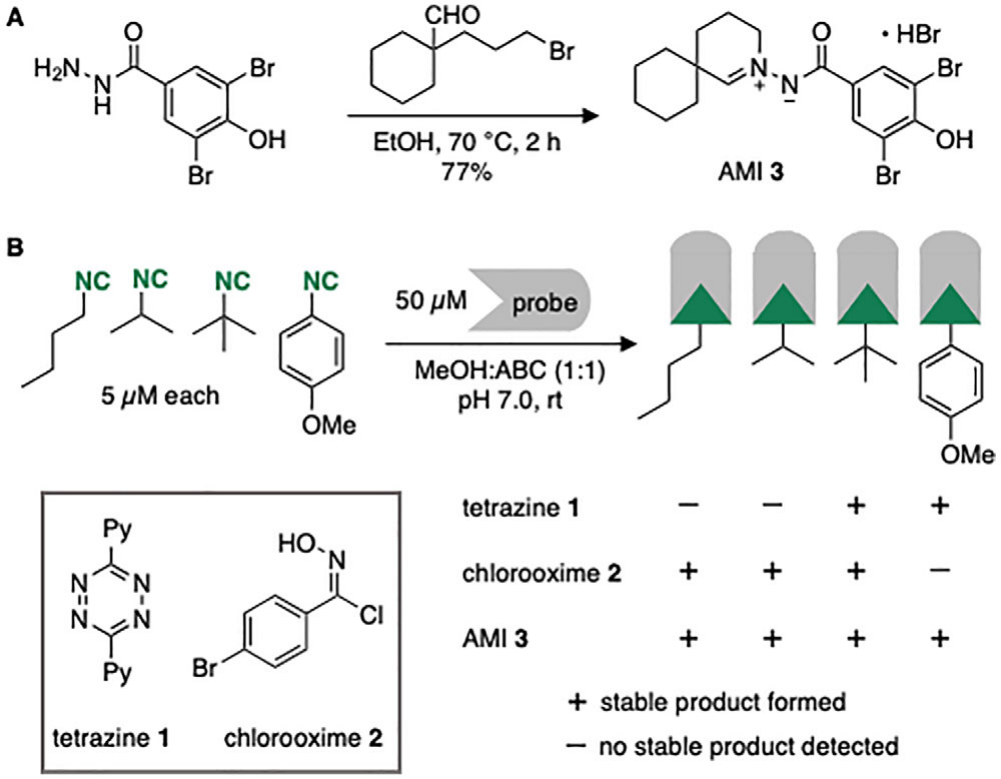
(A) Synthesis of AMI probe **3**. (B) Reaction of a mixture of a primary, secondary, tertiary, and aromatic isonitrile with tetrazine **1**, chlorooxime **2**, or AMI **3**. HPLC‐HRMS analyses showed that only AMI probe **3** formed a stable ligation product with all four isonitriles.

We initiated our studies by designing and synthesizing AMI **3** bearing a dibromide tag to enhance MS‐based analysis of natural product conjugates (Figures 1D and 2A). The dibromide tag generates a distinct isotope pattern and is key for the detection of low‐abundance signals in complex mass spectra [24]. The 4‐hydroxy group on the phenacyl ring was introduced to a) improve water solubility, b) enhance the probe's UV‐Vis absorbance, and c) increase the reactivity of the AMI towards isonitriles [23]. The synthesis of probe **3** was straightforward by condensation of the corresponding aryl hydrazide and 1,5‐bromoaldehyde (Figure 2A, Scheme S1).

To explore the value of **3** as an RBS probe, we used *Escherichia coli* cell lysates (1 mg/mL), suspended in a mixture of methanol (MeOH) and ammonium bicarbonate (ABC) buffer (1:1; pH 7.0), as a mimic of a complex biological matrix. The volatile ABC buffer was selected for its compatibility with LC‐MS analysis [25]. To probe for the detection of different types of isonitriles, the cell lysate was supplemented with a primary, secondary, tertiary, and aromatic isonitrile (*n*‐butyl isocyanide, isopropyl isocyanide, *tert*‐butyl isocyanide, and 4‐methoxyphenyl isocyanide; 5 µM each). Upon addition of AMI **3** (50 µM), the expected stable ligation product formed with each of the tested isonitriles, as evidenced by HPLC‐HRMS analysis (Figure 2B). In each of the four reactions, the *m*/*z* value corresponding to the expected [M+H]^+^ ion was detected (Figures S1–S4). Comparative experiments with chlorooxime probe **2** under the same conditions yielded signals corresponding to the [M+H]^+^ ions of the three aliphatic isonitrile conjugates, albeit at lower intensities (Figures 2B and S1–S4). The ligation product with the aromatic isonitrile, however, was not detected. Consistent with previous reports [12, 13, 14, 15, 16], tetrazine probe **1** only formed stable products with the tertiary and aromatic isonitriles, but not with the primary and secondary isonitriles (Figures S1–S4). Since this probe does not bear a bromide label, detection was difficult. Overall, these studies with model isonitriles in cell lysates demonstrate the value of AMI‐based probe **3** for detecting a broad range of different isonitriles. The results also showcase the benefits of AMI‐based probe **3** over previously developed isonitrile‐targeting RBS probes.

Next, we evaluated the sensitivity of probe **3** for detecting isonitriles. Solutions of *tert*‐butyl isocyanide at different concentrations (ranging from 16 µM to 125 nM) were incubated with probe **3** (50 µM) in MeOH:ABC buffer (1:1; pH 7.0), for 4 h at room temperature. Subsequent HPLC‐HRMS analyses revealed a concentration‐dependent signal of the [M+H]^+^ ion corresponding to the ligation product and identified a detection limit as low as 500 nM (Figure S5). Noteworthy, in contrast to the model isonitrile, many natural products possess functional groups that enhance electrospray ionization efficiency. The actual detection limit of most natural isonitriles is, therefore, most likely even lower.

### 2.2 Evaluation of the AMI Probe for the Identification of Isonitriles in Bacterial Extracts

**FIGURE 3:**
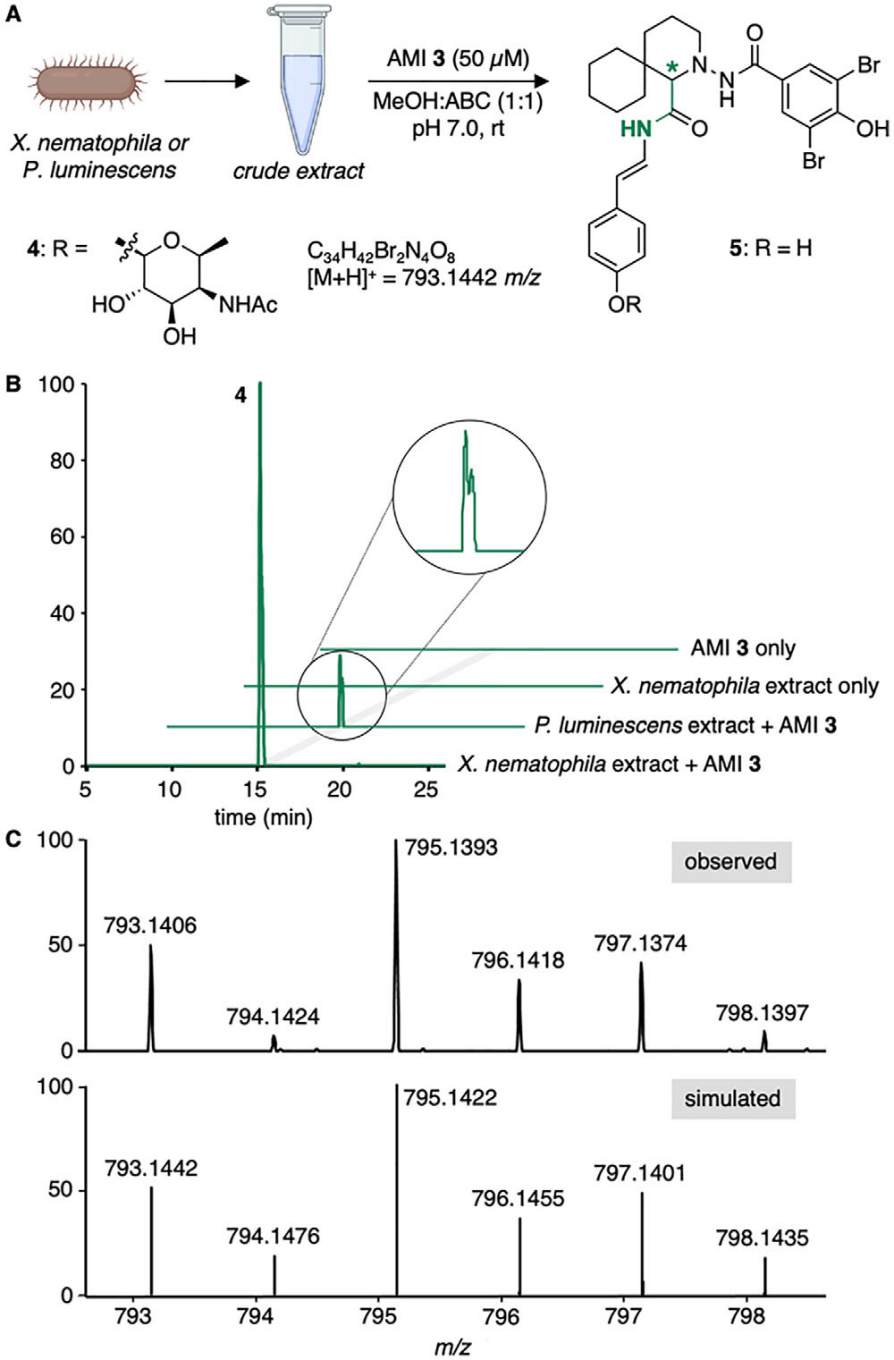
Detection of rhabduscin and its aglycone from bacterial extracts. (A) Treatment of extracts (10 mg/mL) of *X. nematophila* and *P. luminescens* with AMI probe **3**. (B) Extracted ion chromatograms (EICs) of *m*/*z* 793.1442 (**4**), within a mass error of 5 ppm, for extracts of *X. nematophila* and *P. luminescens* with probe **3** and controls with untreated *X. nematophila* extract and probe **3** only. (C) Observed (top) and simulated (bottom) isotope pattern for the [M+H]^+^ ion of rhabduscin ligation product **4**; *m*/*z* calculated for C_34_H_43_Br_2_N_4_O_8_^+^: 793.1442 [M+H]^+^; found 793.1406, Δ *m*/*z*  = −4.5 ppm. For the data of aglycone ligation product **5**, see Figure S6.

Building on these results, we investigated whether probe **3** can identify isonitriles produced by bacteria. *Photorhabdus luminescens* TT01 and *Xenorhabdus nematophila* ATCC19061 are well‐studied entomopathogenic bacterial symbionts of nematodes that produce rhabduscin, a glycosylated isonitrile‐containing natural product (Figure 1A, left), along with its aglycone derivative [10, 26]. To assess the probe's applicability and effectiveness, crude extracts (10 mg/mL) from both bacterial strains were incubated with probe **3** (50 µM) in MeOH:ABC buffer (1:1; pH 7.0) for 4 h at room temperature (Figure 3A). Reassuringly, HPLC‐HRMS analyses revealed [M+H]^+^ ion signals corresponding to the ligation products of AMI **3** with rhabduscin (**4**) and its aglycone (**5**) in both samples (Figures 3B,C, and S6). Control experiments containing only probe **3** or only the crude extracts did not produce these signals (Figure 3B). Notably, the extracted ion chromatograms (EICs) of the ligation product of probe **3** with the chiral rhabduscin (**4**) feature a double peak, consistent with the formation of two diastereomeric products through the newly formed stereogenic center within the ligation product. Thus, RBS probe **3** provides a signature for the occurrence of isonitriles through the dibromide MS isotope pattern and another signature for the occurrence of chiral isonitriles through the emergence of double peaks in chromatograms on achiral stationary phases.

### 2.3 A Unique MS and Chromatography Signature—Discovery of So Far Unknown Isonitriles in Fungi

We moved on to explore whether AMI probe **3** can identify yet unknown isonitrile‐containing natural products. A recent genome mining study by Nickles et al. identified isonitriles as the fifth largest class of specialized metabolites in fungi, after polyketides, nonribosomal peptides, terpenes, and alkaloids [2]. This finding underscores the importance of isonitrile‐containing natural products in the fungal kingdom. To identify candidate fungi that contain genes encoding isocyanide synthases, we performed a standard Position‐Specific Iterated Basic Local Alignment Search Tool (PSI‐BLAST) search to find distant evolutionary relationships among protein sequences. The bacterial isocyanide synthase IsnA from the rhabduscin‐producing *X. nematophila* served as a query to search for fungal genome sequences in the National Center of Biotechnology Information (NCBI) repository. This search yielded several fungi encoding at least one Dit1‐like protein, enzymes that are distant sequence homologs of IsnA implicated in fungal sporulation [27, 28]. In *Saccharomyces cerevisae*, Dit1 catalyzes the formation of *N*‐formyl tyrosine with the postulated involvement of an isonitrile intermediate [27, 28]. *N*‐formyl tyrosine is subsequently converted into *N,N’*‐bisformyl dityrosine, which contributes to spore wall maturation in *S. cerevisiae* [27, 29]. Among the identified fungi encoding Dit1‐like proteins, we selected *Aspergillus nidulans* FGSC A4 and *Phycomyces blakesleeanus* ATCC 8743b, strains available in our labs, for further studies with AMI probe **3**.

**FIGURE 4:**
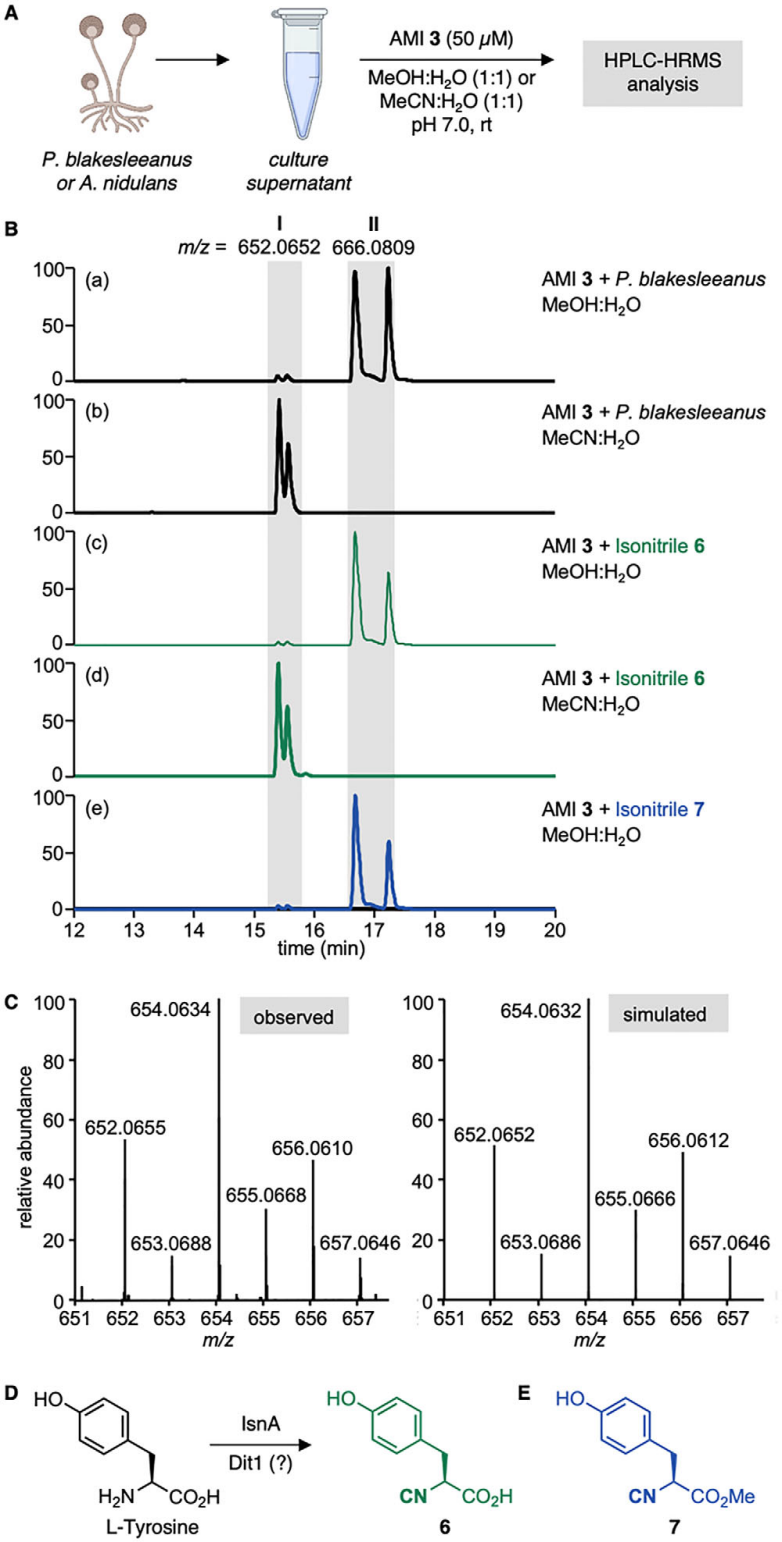
Identification of isonitrile **6** from a fungal sample. (A) Treatment of culture supernatants of *A. nidulans* and *P. blakesleeanus* with AMI probe **3**. (B) Extracted ion chromatograms (EICs) of *m*/*z* 652.0652 and 666.0809, within a mass error of 5 ppm, corresponding to **I** and **II**, of HPLC‐HRMS chromatograms. Black: Probe **3** with *P. blakesleeanus* supernatant in (a) MeOH:H_2_O and (b) MeCN:H_2_O. Green and blue: Probe **3** with synthetic isonitriles **6** (c and d) and **7** (e) in either MeOH:H_2_O or MeCN:H_2_O. (C) Observed (left) and simulated (right) isotope pattern for signal **I**. *m/z* calculated for C_27_H_32_Br_2_N_3_O_6_^+^: 652.0652 [M+H]^+^; found 652.0655, Δ *m*/*z* = 0.5 ppm. (D) Postulated biosynthesis of isonitrile **6**. (E) Methylester **7**.

Liquid cultures of *A. nidulans* and *P. blakesleeanus* were grown for five days in cornmeal medium. The supernatants were then incubated with probe **3** (50 µM) in MeOH:H_2_O (1:1; pH 7.0), followed by HPLC‐HRMS analysis (Figure 4A). Control experiments were performed without the probe and without the fungal supernatants. The data were then processed with the Thermo Compound Discoverer 3.2 analysis software, utilizing the probe's dibromide isotope pattern for identification of isonitrile conjugates.

In the mass spectra from *A. nidulans*, no new signals with a dibromide isotope pattern emerged from incubation with probe **3**. In contrast, the sample from *P. blakesleeanus* contained two new signals: **I** (*m*/*z* =  652.0655) and **II** (*m*/*z* =  666.0794), both appearing as double peaks (Figures 4Ba and S7). Signal **II** is significantly more pronounced than signal **I**, with a mass difference of +14, indicative of an additional CH_2_ group. Signal **I** matches the simulated [M+H]^+^ ion arising from the ligation of AMI **3** with the tyrosine‐related isonitrile **6** (Figures 4C,D). In bacteria, this isonitrile natural product is synthesized from l‐tyrosine by IsnA (Figure 4D) [26], but **6** has so far not been identified in fungi [28].

To analyze whether signal **I** arises indeed from tyrosine‐related isonitrile **6**, we synthesized an authentic standard of **6** in two steps from l‐tyrosine methylester (Scheme S2). Incubation of the authentic standard **6** with AMI probe **3** (both 50 µM) in MeOH:ABC buffer (1:1; pH 7.0), resulted in the formation of two signals with identical HPLC retention times and MS features as signals **I** and **II** (Figure 4Bc). This finding corroborates that the fungus *P. blakesleeanus* produces isonitrile **6**. The result also implies that, contrary to a previous report [30], the isonitrile formed by Dit1 is not merely an intermediate *en route* to *N*‐formyl tyrosine but a stable natural product. Notably, isonitrile **6** is the first isonitrile identified from the Mucoromycota phylum, fungal species in which isocyanide synthases are scarce [2, 31]. Further MS/MS analysis validated that signal **I**, obtained from the fungal sample and AMI probe **3**, is identical to the analogous signal derived from the authentic standard **6** and probe **3** (Figure S8). Consistent with the emergence of diastereoisomers upon reaction of chiral, enantiomerically pure isonitrile **6** with AMI probe **3**, the ligation product appears as two signals. These results further corroborate the unique dual signature of AMI probe **3** for the identification of chiral and achiral isonitriles.

### 2.4 Participating and Nonparticipating Solvents Provide a Signature for Isonitriles Bearing an α‐carboxylic Acid

Next, we investigated the origin of the compound corresponding to signal **II** with a mass of +14 compared to signal **I**. We suspected that this compound arises from a reaction with MeOH, the solvent used to ensure solubility during the RBS. Indeed, incubation of *P. blakesleeanus* supernatant or an authentic standard of **6** with probe **3** in MeCN instead of MeOH as the organic cosolvent yielded signal **I**, but not signal **II** (Figure 4Bb,Bd).

This solvent incorporation was unexpected, as in all previous reactions of AMIs with isonitriles — including the four model isonitriles (Figure 2B), rhabduscin, and its aglycone (Figure 3A) — MeOH incorporation into the ligation product was not observed. To investigate the location of the additional CH_2_ group, we synthesized methylester **7** (Figure 4E, Scheme S2). Reaction with AMI probe **3** and HPLC‐HRMS analysis provided a signal identifical to signal **II** (Figure 4Be). This finding implies that signal **II** corresponds to the methylester of signal **I**.

**FIGURE 5:**
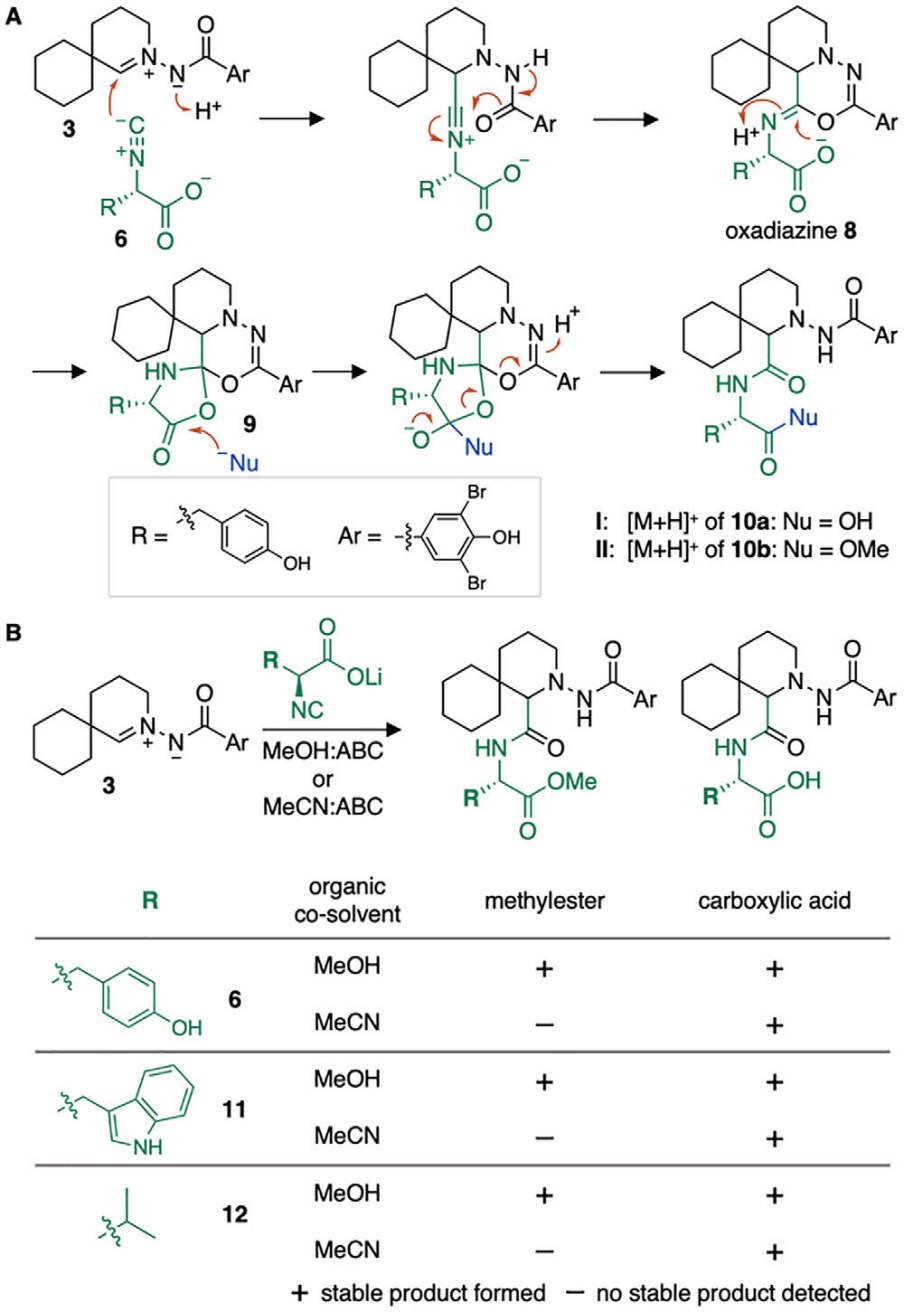
Reactivity of probe **3** toward isonitriles bearing an α‐carboxylic acid. (A) Mechanistic proposal for the incorporation of MeOH into ligation product **10b**. (B) Reaction of AMI probe **3** with isonitriles **6**, **11**, and **12**. In all cases, signals corresponding to the [M+H]^+^ ions of both ligation products—free carboxylic acid (right) and methylester (left)—were observed by HPLC‐HRMS analysis, when the reaction was conducted in MeOH:buffer. In MeCN:buffer, only signals corresponding to the [M+H]^+^ ions of the carboxylic acid ligation products (right) were detected.

We therefore reasoned that the carboxylic acid group α to the isonitrile participates in the reaction. The AMI‐isonitrile ligation proceeds through an oxadiazine intermediate that subsequently hydrolyses to form the final amide product (Figure 1C) [22, 23]. It is plausible that a carboxylate α to the parent isonitrile group outcompetes intermolecular solvolysis and reacts intramolecularly with oxadiazine **8** to form spiro‐oxazolidinone **9** (Figure 5A). Subsequent nucleophilic addition of either water or MeOH, followed by fragmentation of the spirocycle, furnishes carboxylic acid **10a** or methylester **10b**, respectively.

The formation of **10b** was further confirmed by synthesis from isonitrile **7** and AMI **3** with structural analysis by NMR spectroscopy, X‐ray crystallography, and HRMS. HPLC‐HRMS analyses validated the identity between chemically synthesized **10b** and **II** (Figure S9).

Methylester formation of isonitriles bearing an α‐carboxylic acid can be useful for isonitrile analyses and also in organic synthesis. We, therefore, evaluated the generality of the method and synthesized tryptophan‐ and valine‐derived isonitriles **11** and **12**, to our knowledge the only other reported natural amino acid‐derived isonitriles [32, 33]. HPLC‐HRMS analyses of reactions between **11** (50 µM) or **12** (50 µM) with probe **3** (50 µM) in MeCN:ABC buffer (1:1; pH 7.0) only detected signals corresponding to the [M+H]^+^ ion of the ligation products with a carboxylic acid. In contrast, in MeOH:ABC buffer (1:1; pH 7.0), both the carboxylic acid and the corresponding methylester derivatives formed, mirroring the results obtained with tyrosine‐derived isonitrile **6** (Figures 5B, and S10, S11). All signals appeared as double peaks, indicative of the formation of diastereomeric products. These findings show that methylester formation is a general feature of isonitriles bearing an α‐carboxylic acid and a useful diagnostic tool.

For the agnostic detection of natural isonitriles in a biological matrix, we thus propose a workflow in which the crude biological sample is incubated with AMI probe **3** under two parallel conditions: in MeOH:aqueous buffer and in MeCN:aqueous buffer. A subsequent, ideally bioinformatics‐assisted, MS‐analysis, employing the characteristic isotope pattern of the dibromide MS‐tag and comparison to control samples, will reveal whether the sample contains isonitriles. The presence of a double peak with identical *m*/*z* indicates a chiral parent isonitrile. Moreover, an additional signal corresponding to a mass shift of +14 (CH_2_ group) in the sample containing MeOH hints strongly at an isonitrile featuring a proximal carboxylic acid.

### 2.5 Antibacterial Activity

**FIGURE 6:**
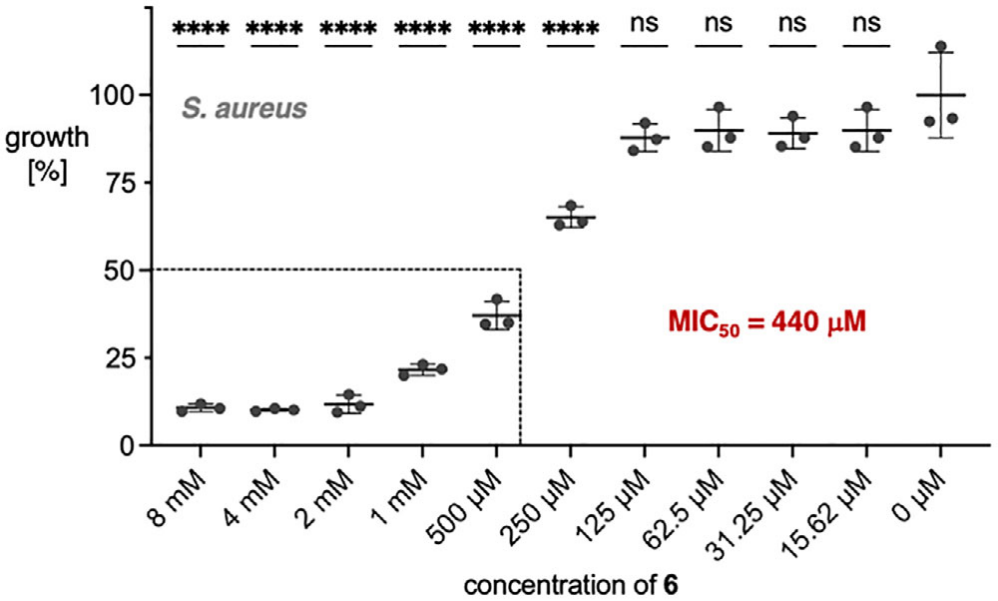
Antibiotic activity of **6** against *S. aureus*. MIC_50_ values were determined after 24 h. The error bars represent the mean ± SD from three independent biological replicates. A one‐way ANOVA with Dunnett's multiple comparisons test was conducted to determine whether bacterial growth differed significantly across the tested concentration range compared to growth in medium alone. Statistical significance was set at *p* < 0.05. **** *p* < 0.0001; ns, not significant.

Finally, we tested the identified tyrosine‐related isonitrile **6** for antibacterial activity. **6** inhibits 50% bacterial growth at minimal concentrations (MIC_50_) in the low millimolar regime (3.3–15 mM) against *E. coli*, *Salmonella enterica*, *Bacillus subtilis*, and a multi‐antibiotic‐resistant strain of *Stenotrophomonas maltophilia* (Figures S12,S13 and Table S1). Against the human pathogen *Staphylococcus aureus*, isonitrile **6** features a MIC_50_ of 440 µM (Figure 6). These findings align with previous reports on the antibacterial properties of fungal isonitrile natural products and underscore their potential for the development of new antibiotics [32, 34].

## 3 Conclusions

In conclusion, we demonstrate the value of the AMI‐isonitrile ligation for the reactivity‐based screening of natural isonitriles. The AMI probe forms stable ligation products with primary, secondary, tertiary, and aromatic isonitriles. The probe selectively detects isonitriles, even at nanomolar concentrations, and a distinct dibromide isotope pattern facilitates a reliable MS‐based identification of the natural product conjugates. The developed workflow further reveals chirality and the presence of an α‐carboxylic acid in the isonitrile natural product. Application of the probe to complex biological matrices detected the known bacterial natural product rhabduscin and identified a postulated, but so far unidentified, fungal isonitrile. Tyrosine‐related isonitrile **6** is, to the best of our knowledge, the first isonitrile‐containing natural product identified from a member of the phylum Mucoromycota, comprising some of the earliest diverging fungal lineages. Taken together, the herein developed AMI probe represents a powerful, highly selective, and general tool for the discovery of natural isonitriles, with exciting prospects for the development of novel bioactive compounds.

## Conflicts of Interest

The authors declare no conflict of interest.

## Supporting information



The authors have cited additional references within the Supporting Information.

**Supporting File 1**: chem70671‐sup‐0002‐Data.zip.

## Data Availability

The data that support the findings of this study are available in the supplementary material of this article.
