# Supplementary material for: Identification of Natural Isonitriles Through Ligation to an Azomethine Imine Probe
Source: Chemistry. 2026 Jan 15;32(11):e03642. doi: 10.1002/chem.202503642 (PMC13005658; doi:10.1002/chem.202503642)

Supporting Information

Identification of Natural Isonitriles Through Ligation to an Azomethine Imine Probe

Maurice P. Biedermann,[a] Alexander Brachmann,[b] Shurui Mai,[b] Athanasios Markos,[a,c] Markus Künzler,[b] Jörn Piel,[b] and Helma Wennemers*[a]

[a] Laboratory of Organic Chemistry, D-CHAB, ETH Zürich, Vladimir-Prelog-Weg 3, 8093 Zürich, Switzerland
[b] Institute of Microbiology, D-BIOL, ETH Zürich, Vladimir-Prelog-Weg 4, 8093 Zürich, Switzerland
[c] Current address: Department of Organic Chemistry, Faculty of Science, Palacký University, 779 00 Olomouc, Czech Republic

E-mail: helma.wennemers@org.chem.ethz.ch

Table of Contents

1 General aspects **S3**

2 Experimental procedures **S3**

2.1 High-performance liquid chromatography–mass spectrometry (HPLC–MS) analysis S3

2.2 Computational MS data analysis S4

2.3 Bacterial strains and media S4

2.4 Fungal strains and media S4

2.5 Reaction of the three isonitrile RBS screening probes with four model isonitriles S5

2.6 LOD determination S8

2.7 General procedure for the labeling of isonitriles from bacterial extracts with probe **3** S9

2.8 Detection of rhabduscin and its aglycone S9

2.9 Procedure for the RBS of fungal isonitriles with probe **3** S10

2.10 MS/MS analysis of **I** S11

2.11 Comparison of chemically synthesized **10b** with signal **II** obtained from incubation of AMI probe **3** with *P. blakesleeanus* S12

2.12 Procedure for the reaction of synthetic isonitriles with AMI probe **3** S12

2.13 Reaction between AMI **3** and tryptophan- and valine isonitriles **11** and **12** S13

2.14 Antibacterial assays S14

2.15 UV-Vis absorption spectrum of probe **3** S16

3 Synthetic procedures **S17**

3.1 Synthesis of probe **3** S17

3.2 Synthesis of isonitrile **6** S18

3.3 Synthesis of ligation product **10b** S19

***4 X-ray crystallographic data* S20**

***5 PSI-BLAST query* S21**

***6 References cited only in the Supporting Information* S21**

***7 ^{1}H and ^{13}C NMR spectra* S22**

1 General aspects

All purchased chemicals were used as received. Reaction mixtures were stirred magnetically. Air- and moisture-sensitive liquids and solutions were transferred via syringe into the reaction vessels through rubber septa. Yields refer to spectroscopically pure compounds unless otherwise stated. Flash chromatography was performed on silica gel (Merck Kieselgel 60 F254 230–400 mesh). Thin layer chromatography (TLC) was performed on glass-backed silica plates (Merck, Silica Gel 60 F254). **NMR** Spectra were recorded on 400, 500, or 600 MHz instruments. Chemical shifts (δ) are quoted in parts per million (ppm) and referenced to the appropriate NMR solvent peak(s). Coupling constants (*J*) are reported in Hertz. **HRMS**: High resolution mass spectra were recorded on an ESI-Q-TOF or an EI-Sector-MS. **UV-Vis** absorption was measured on a Cary 300 spectrometer. Crystals suitable for X-ray analysis were selected and measured on a XtaLAB Synergy R, HyPix-Arc 150 diffractometer.

2 Experimental procedures

2.1 High-performance liquid chromatography–mass spectrometry (HPLC–MS) analysis

All reactivity-based screening samples were analyzed by HPLC coupled to high-resolution heated electrospray ionization MS (HPLC–HR–HESI–MS). Data were acquired on either a Thermo Scientific™ Orbitrap Ascend Tribrid Mass Spectrometer coupled to a Thermo Vanquish Flex UHPLC system or a Thermo Scientific™ Q Exactive Orbitrap coupled to a Thermo Dionex UltiMate 3000 system. The following chromatographic and MS parameters were used:

<u>Instrument 1: Thermo Orbitrap Ascend</u>

- Column: Phenomenex Kinetex XB-C18 (2.6 µm, 150 × 4.6 mm)
- Mobile phases: A = H_2O + 0.1% formic acid; B = acetonitrile + 0.1% formic acid
- Flow rate: 0.5 mL/min
- Gradient: 5% B (0–2 min), linear to 98% B (2–20 min), hold at 98% B (20–22 min), return to 5% B (22–22.5 min), re-equilibration at 5% B (22.5–25 min)
- Ionization mode: Positive HESI
- Scan range: m/z 100–1200
- Resolution: 120,000 (at m/z 200)
- Spray voltage: 3.5 kV
- Sheath gas: 60 (arb. units)
- Auxiliary gas: 15 (arb. units)
- Sweep gas: 2 (arb. units)
- Ion transfer tube temperature: 350 °C
- Vaporizer temperature: 350 °C
- RF lens: 40%
- AGC target: 4×10^5
- Maximum injection time: 50 ms
- Microscans: 1

Instrument 2: Thermo Q Exactive Orbitrap

- Column: Phenomenex Kinetex XB-C18 (2.6 µm, 150 × 4.6 mm)
- Mobile phases: A = H_2O + 0.1% formic acid; B = acetonitrile + 0.1% formic acid
- Flow rate: 0.7 mL/min
- Gradient: 5% B (0–2 min), linear to 98% B (2–20 min), hold at 98% B (20–25 min), return to 5% B (25–27 min)
- Ionization mode: Positive HESI
- Scan range: m/z 100–1500
- Resolution: 70,000 (at m/z 200)
- Spray voltage: 3.5 kV
- Sheath gas: 58 (arb. units)
- Auxiliary gas: 16 (arb. units)
- Sweep gas: 3 (arb. units)
- Ion transfer tube temperature: 350 °C
- Vaporizer temperature: 463 °C
- S-lens RF level: 50
- AGC target: 1×10^6
- Maximum injection time: 100 ms
- Microscans: 1

2.2 Computational MS data analysis

To identify potential ligation products between probe **3** and natural isonitriles in crude fungal extracts, data were processed using Thermo Scientific™ Compound Discoverer 3.2. Comparative analysis was performed between samples with and without probe **3**. Candidate features were filtered based on the presence of a characteristic dibromide isotope pattern and corresponding intensity ratios.

2.3 Bacterial strains and media

Xenorhabdus nematophila HGB081 and *Photorhabdus luminescens* TT01 were cultivated in Luria–Bertani (LB) medium composed of yeast extract (10 g L^{-1}), tryptone (5 g L^{-1}), and NaCl (10 g L^{-1}), adjusted to pH 7.0 with distilled water. For solid media, LB was supplemented with agar (20 g L^{-1}). Cultures were incubated at 30 °C with shaking at 180 rpm.

2.4 Fungal strains and media

Corn meal medium (CMM) was prepared by dissolving 8.5 g BBL™ Corn Meal Agar (CMA) powder, 5 g malt extract and 2 mL of 10% KOH (*w/v*) in 1 L deionized water, followed by filtration through a 0.22 µm filter to remove any undissolved agar. The liquid CMM was then autoclaved. Sporangiospores of *Phycomyces blakesleeanus* (ATCC 8743b) or *Aspergillus nidulans* (FGSC A4) were cultivated in unbaffled Erlenmeyer flasks with 100 mL of CMM at 23 °C with shaking at 180 rpm for 5 days. After cultivation, the culture was centrifuged at 12’000 × g for 10 min. The supernatant was collected, flash-frozen in liquid nitrogen, and stored at –80 °C for further analysis.

2.5 Reaction of the three isonitrile RBS screening probes with four model isonitriles

A mixture of isonitriles consisting of *n*-butyl isocyanide, isopropyl isocyanide, *tert*-butyl isocyanide, and 4-methoxyphenyl isocyanide (5 µM each) was dissolved in a 1:1 mixture of MeOH and lyophilized cell lysate from *E. coli* (1 mg/mL), resuspended in ABC buffer (50 mM; pH 7.0). This suspension was incubated with AMI probe **3**, chlorooxime probe **2**, or tetrazine probe **1** (50 µM final concentration) and gently shaken at room temperature for 4 h. Control samples were prepared that contained only the probes (no mixture of isonitriles) or only the mixture of isonitriles (no probes). The suspensions were then filtered and analyzed by HPLC-HRMS. Due to the same molecular weight of *n*-butyl isocyanide and *tert*-butyl isocyanide, these two isonitriles were also incubated with the probes separately to identify the retention times of the corresponding conjugates. For the sample containing AMI probe **3**, both the oxadiazine and the amide ligation products were detected. Conjugates to tetrazine probe **1** eluted very early and usually gave no well-defined peaks. In the sample of tetrazine probe **1** and isopropyl isocyanide, traces of the signal corresponding to the ligation product were detected, but this signal disappeared over time (Figure S3).

	***n*-butyl NC**	***i*-propyl NC**	***t*-butyl NC**	**4-MeOPh NC**
**Structure**	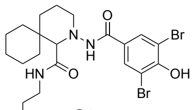 **S1**	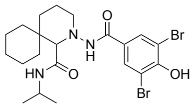 **S2**	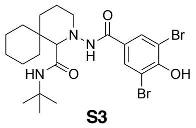 **S3**	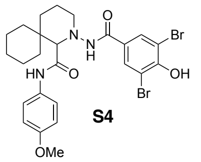 **S4**
m/z [M+H]$^+$	544.0805	530.0648	544.0805	594.0598
rt (min)	18.09	17.17	18.53	18.59 + 18.76
**Structure**	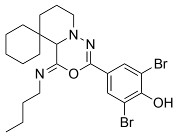 **S5**	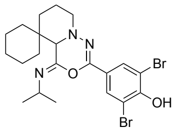 **S6**	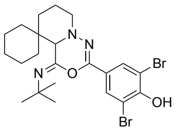 **S7**	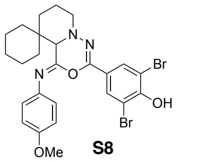 **S8**
m/z [M+H]$^+$	526.0699	512.0543	526.0699	576.0492
rt (min)	24.70	23.10	24.34	23.48 + 23.74
**Structure**	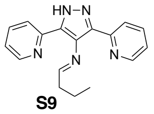 **S9**	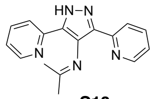 **S10**	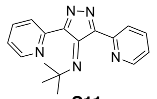 **S11**	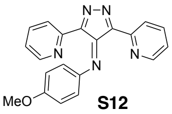 **S12**
m/z [M+H]$^+$	292.1557	278.1400	292.1557	342.1349
rt (min)	*not detected*	2.31	2.4–2.8	2.0–2.2
**Structure**	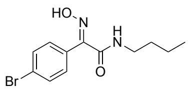 **S13**	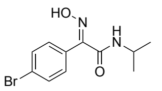 **S14**	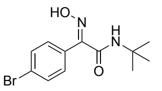 **S15**	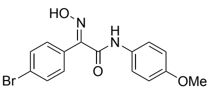 **S16**
m/z [M+H]$^+$	299.0390	285.0233	299.0390	349.0182
rt (min)	14.62	13.06	14.74	*not detected*

Figure S1: Overview of all theoretically possible ligation products between the investigated AMI, tetrazine and chlorooxime probes and the four isonitriles. The calculated *m/z* of the respective [M+H]$^+$ ions and the observed retention times are indicated.

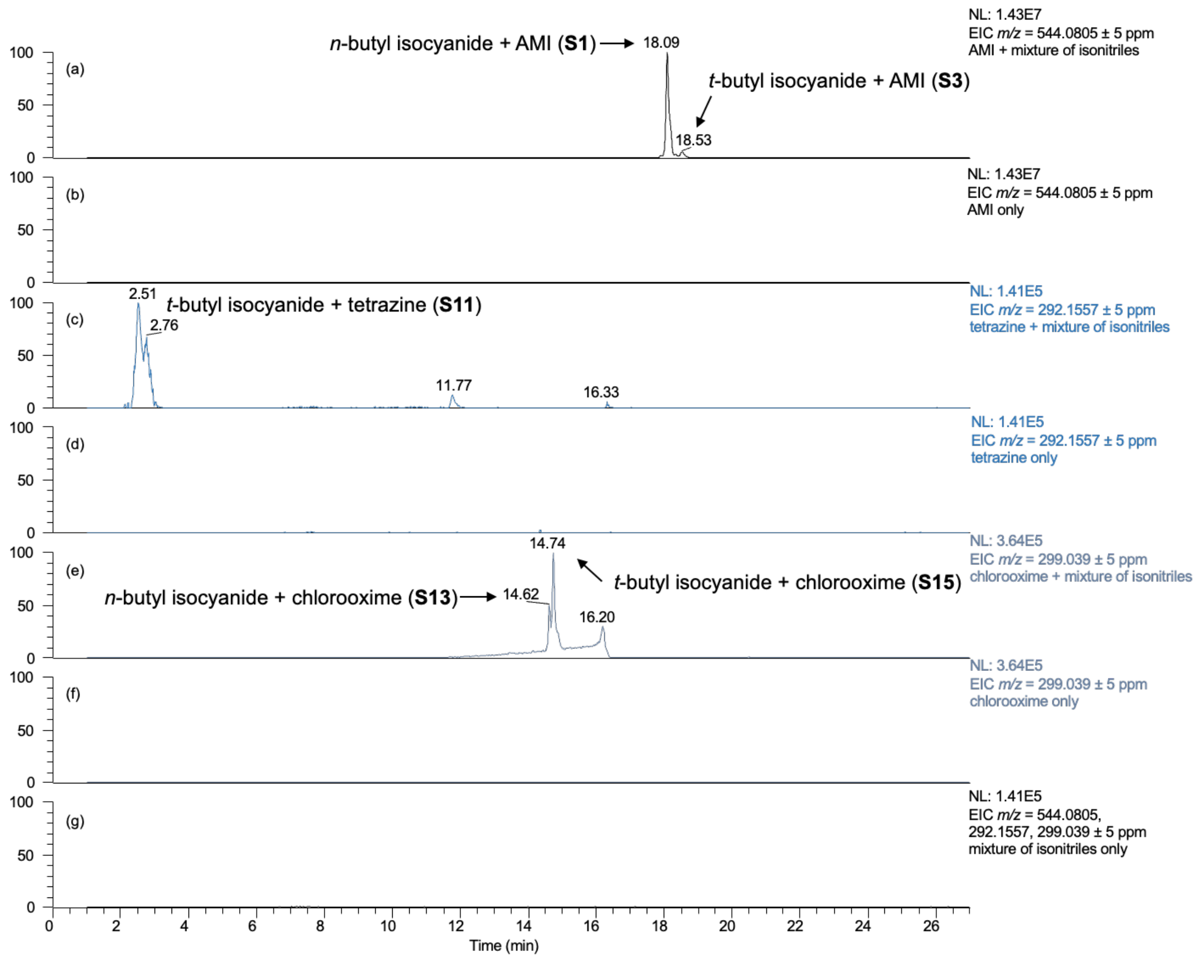

Figure S2: Extracted Ion Chromatograms (EICs) for *m/z* values corresponding to the calculated $[M+H]^+$ ions of ligation products of the three probes to *n*-butyl- and *tert*-butyl isocyanide. (a) and (b): Samples containing AMI probe **3** with (a) and without (b) the mixture of isonitriles. (c) and (d): Samples containing tetrazine probe **1** with (c) and without (d) the mixture of isonitriles. (e) and (f): Samples containing chlorooxime probe **2** with (e) and without (f) the mixture of isonitriles. (g): Control with the mixture of isonitriles alone (no probe added).

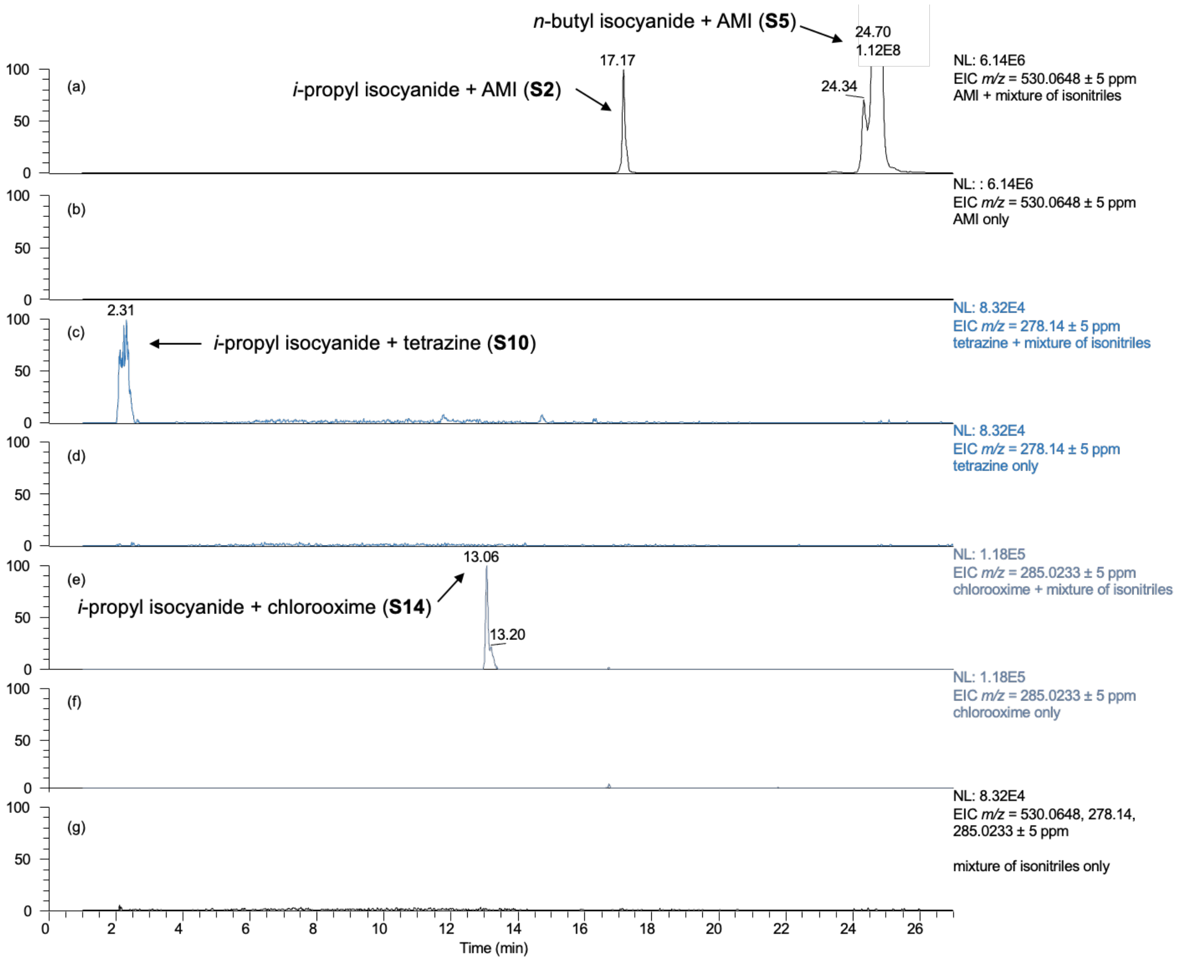

Figure S3: Extracted Ion Chromatograms (EICs) for *m/z* values corresponding to the calculated $[M+H]^+$ ions of ligation products of the three probes to isopropyl isocyanide. (a) and (b): Samples containing AMI probe **3** with (a) and without (b) the mixture of isonitriles. The intense signal at 24.7 min corresponds to the $[M+H]^+$ ion of oxadiazine ligation product to *n*-butyl isocyanide (**S5**). (c) and (d): Samples containing tetrazine probe **1** with (c) and without (d) the mixture of isonitriles. The signal corresponding to the $[M+H]^+$ ion of **S10** (third chromatogram) disappeared after 24 h, consistent with a hydrolytically labile linkage. (e) and (f): Samples containing chlorooxime probe **2** with (e) and without (f) the mixture of isonitriles. (g): Control with the mixture of isonitriles only (no probes added).

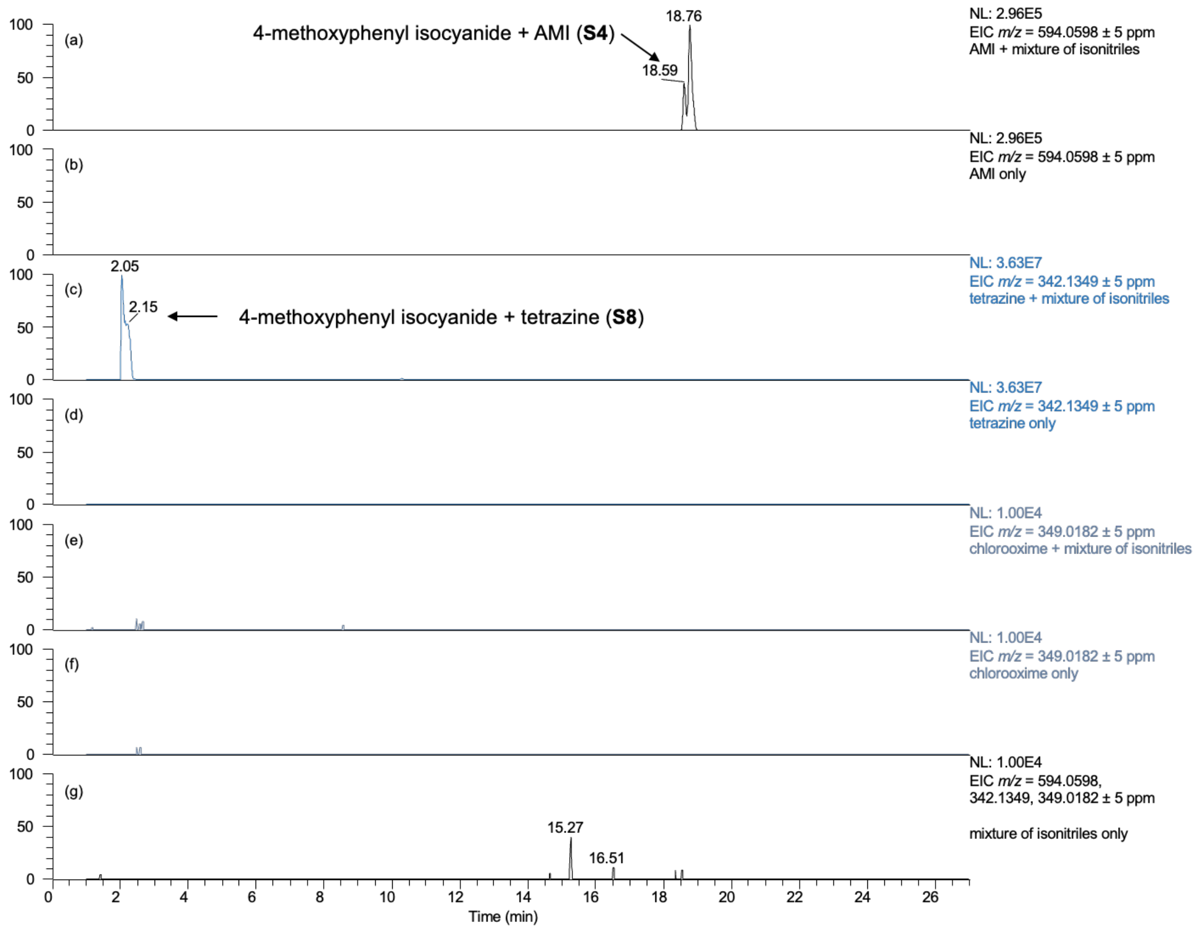

Figure S4: Extracted Ion Chromatograms (EICs) for *m/z* values corresponding to the calculated $[M+H]^+$ ions of ligation products of the three probes to 4-methoxyphenyl isocyanide. (a) and (b): Samples containing AMI probe **3** with (a) and without (b) the mixture of isonitriles. (c) and (d): Samples containing tetrazine probe **1** with (c) and without (d) the mixture of isonitriles. (e) and (f): Samples containing chlorooxime probe **2** with (e) and without (f) the mixture of isonitriles. (g): Control with the mixture of isonitriles only (no probes added).

2.6 LOD determination

The limit of detection (LOD) for the ligation of AMI probe **3** toward different concentrations of *tert*-butyl isocyanide was investigated. The reactions were performed under the following conditions: 50 µM probe **3**, MeOH:ABC buffer (1:1, 50 mM, pH 7.0). A two-fold dilution series of *tert*-butyl isocyanide was created with isocyanide concentrations between 0.125 µM and 16 µM. After incubation at room temperature for 4 h, the samples were quenched with 150 µM KCN prior to filtration and HPLC-HRMS analysis.

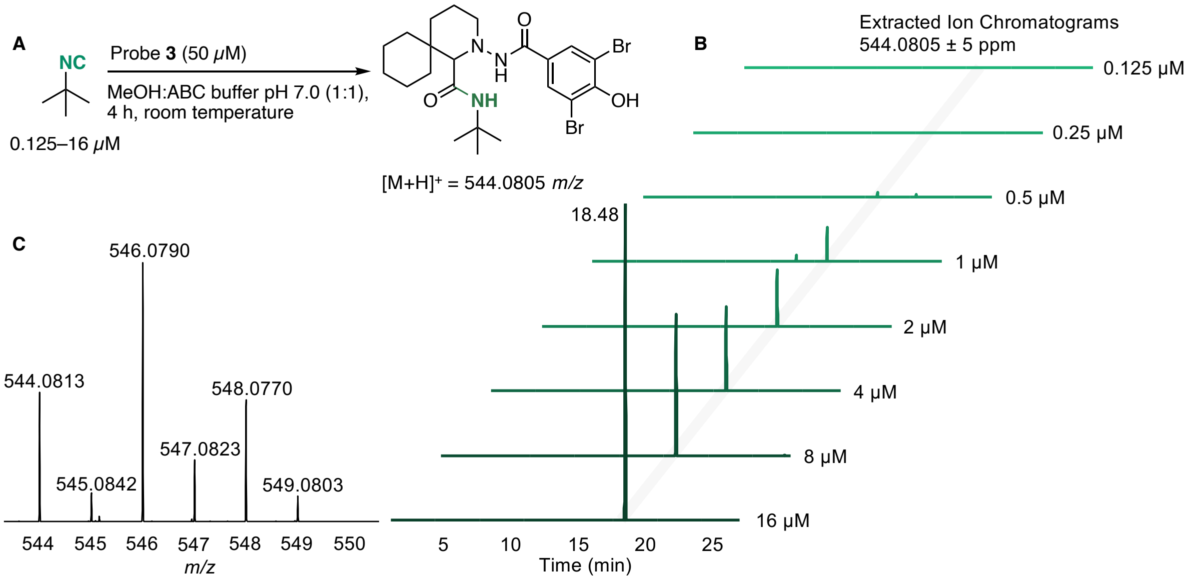

Figure S5: LOD determination for the reaction between probe **3** and *tert*-butyl isocyanide. A: Reaction scheme and conditions. B: Extracted ion chromatograms (EICs) of *m/z* 544.0805 ± 5 ppm, corresponding to the calculated [M+H]+ ion of the ligation product, across the *tert*-butyl isocyanide dilution series. Peak intensities are shown on a uniform scale. C: Isotope pattern of the ligation product, displaying the characteristic dibromide signature.

2.7 General procedure for the labeling of isonitriles from bacterial extracts with probe 3

Bacterial extracts were dissolved in freshly prepared ABC buffer (50 mM; pH 7.0) in an Eppendorf tube. AMI probe **3** was added from a methanolic stock solution to obtain final concentrations of 10 mg/mL bacterial extract, 50 µM probe **3**, and 1:1 MeOH:buffer. The Eppendorf tube was gently shaken at room temperature for 4 h and the mixture was then filtered and subjected to HPLC-HRMS analysis.

2.8 Detection of rhabduscin and its aglycone

Treatment of an extract of *X. nematophilia* ATCC19061 (10 mg/mL) with probe **3** (50 µM) in MeOH:ABC buffer (pH 7.0; 1:1) for 4 h and subsequent HPLC-HRMS analysis revealed signals corresponding to the calculated [M+H]+ ions of the ligation product with rhabduscin (**4**, *m/z* 793.1142) and its aglycone (**5**, *m/z* 606.0598).

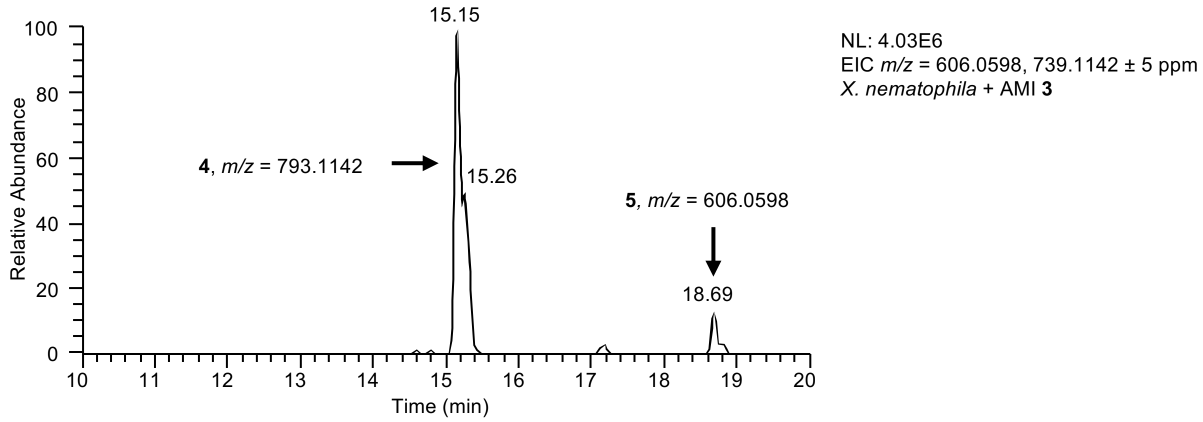

Figure S6: Extracted ion chromatogram (EIC) for *m/z* 606.0598 and 793.1142 ± 5 ppm.

2.9 Procedure for the RBS of fungal isonitriles with probe 3

Supernatants from *Phycomyces blakesleeanus* and *Aspergillus nidulans* were adjusted to pH 7.0 with 1 M HCl. AMI probe **3** (dissolved in MeOH or MeCN) was added to reach a 1:1 ratio of water:organic solvent and a concentration of 50 µM of **3**. After incubation at room temperature overnight, the samples were filtered and analyzed by HPLC-HRMS. Controls were performed with supernatant only or probe **3** only. Signals **I** and **II** were only visible when both the supernatant and probe **3** were present. The small signal at 15.41 min in the top chromatogram has a different retention time than both **I** and **II** and lacks the dibromide isotope pattern.

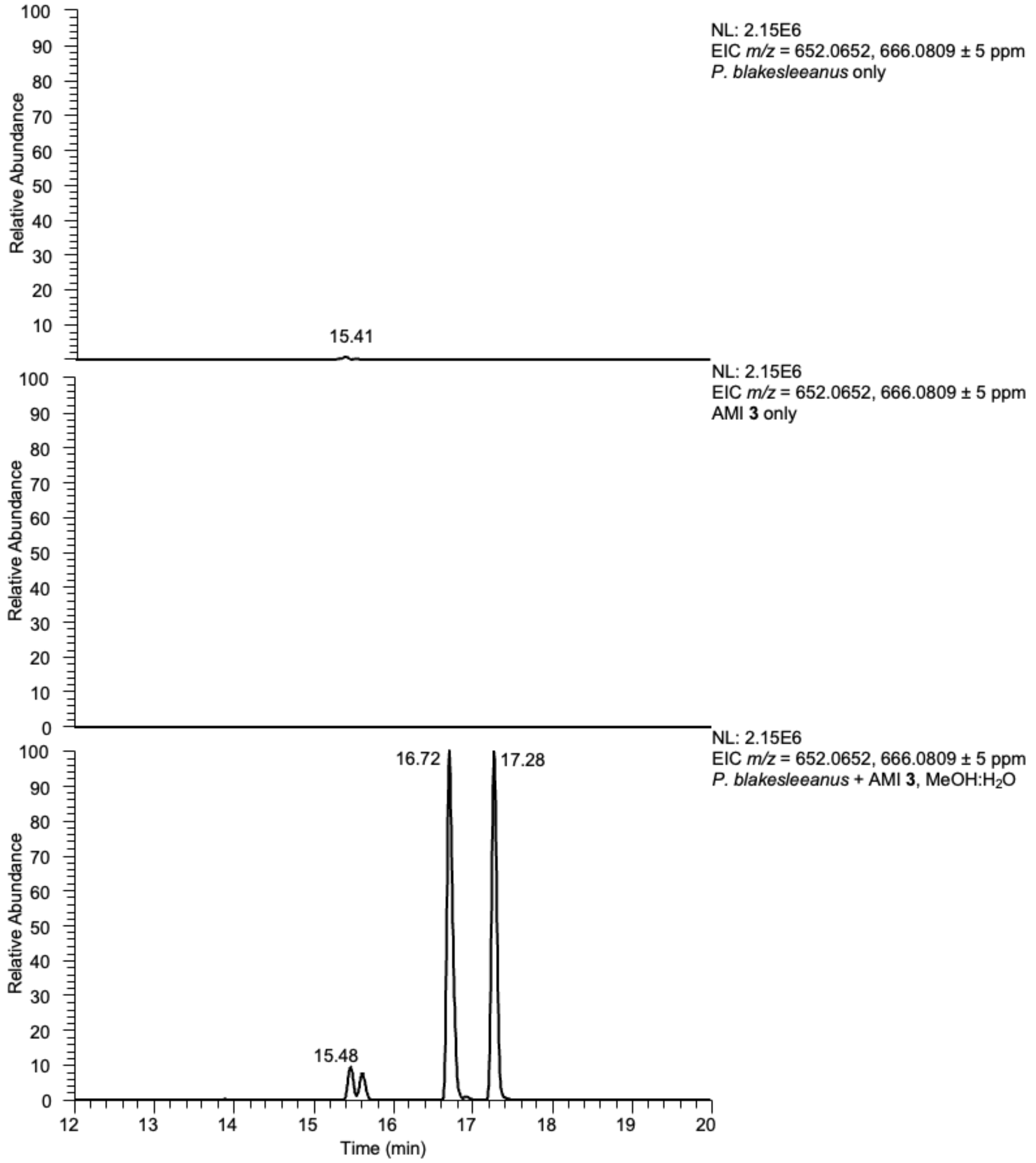

Figure S7: Extracted ion chromatograms (EICs) for *m/z* 652.0652 and 666.0809 ± 5 ppm, corresponding to signals **I** and **II**, respectively, for the incubation of *P. blakesleeanus* supernatant with AMI probe **3** in MeOH:water (1:1, pH 7.0) (bottom) and controls with probe **3** only (middle) and *P. blakesleeanus* supernatant only (top).

2.10 MS/MS analysis of I

The *m/z* 652.0652 corresponding to signal **I**, i.e., the $[M+H]^+$ ion of **10a**, was subjected to MS/MS fragmentation. This MS/MS analysis was performed with HPLC-MS samples from a) incubation of AMI **3** (50 µM) with authentic standard **6** (50 µM, Figure S8, top) and b) incubation of AMI **3** (50 µM) with the supernatant of *P. blakesleeanus* (Figure S8, bottom), both with 50% MeCN as organic co-solvent.

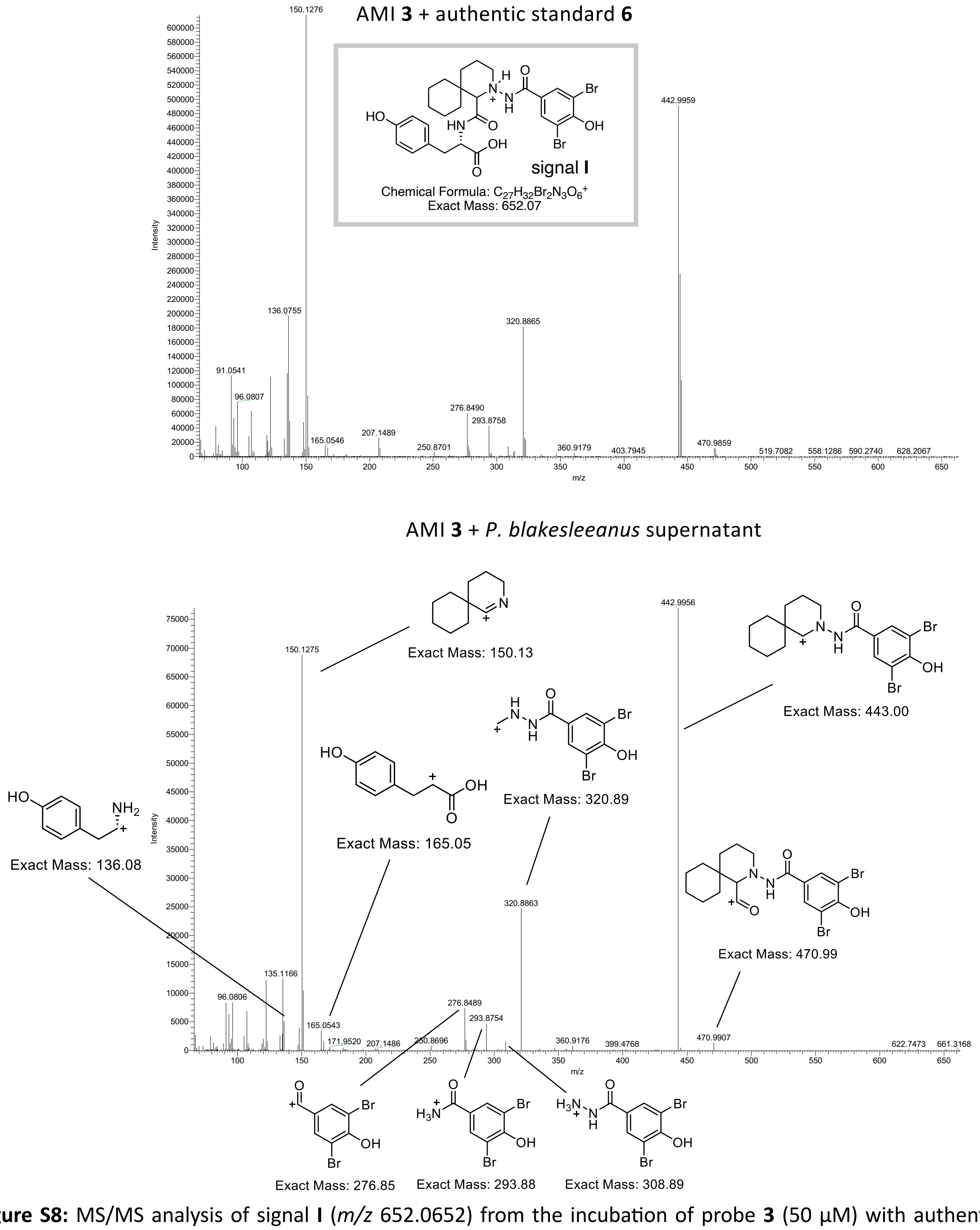

Figure S8: MS/MS analysis of signal **I** (*m/z* 652.0652) from the incubation of probe **3** (50 µM) with authentic standard **6** (50 µM) in MeCN:ABC buffer (1:1, pH 7.0, top) and with supernatant from *P. blakesleeanus* in MeCN:water (1:1, pH 7.0, bottom).

2.11 Comparison of chemically synthesized 10b with signal II obtained from incubation of AMI probe 3 with *P. blakesleeanus*

Chemically synthesized **10b** was compared to signal **II** by HPLC-HRMS analysis. The matching retention times, *m/z* ratios, and isotope patterns suggest that the two compounds are identical. The different ratio of the diastereoisomers can be attributed to enrichment of one diastereoisomer during purification of chemically synthesized **10b**.

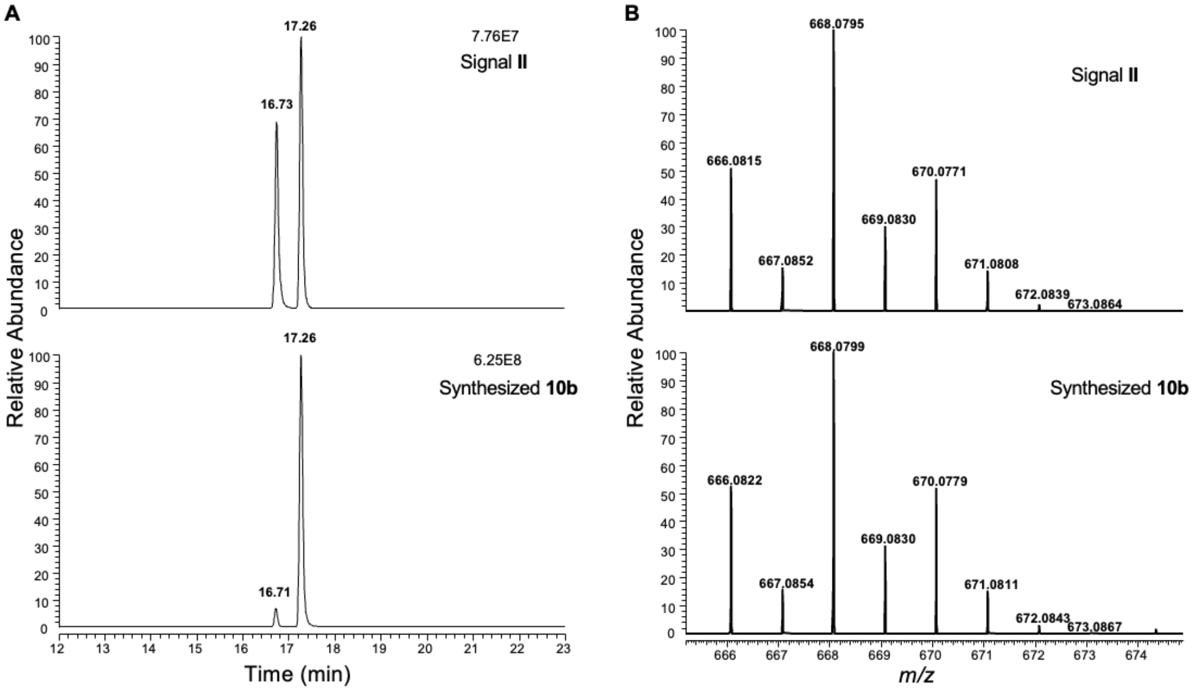

Figure S9: Ligations product from synthetic **10b** and from *P. blakesleeanus*. A) Extracted ion chromatograms (EICs) for *m/z* 666.0809 ± 5 ppm for signal **II**, derived from the fungal supernatant + AMI probe **3**, in water:MeOH (1:1, top) and the synthetic ligation product **10b** (bottom), displaying identical retention times. B) The corresponding isotope patterns for the detected ion at 17.26 min, for the fungal sample *m/z* 666.0815, Δ *m/z* = 0.9 ppm (top) and the synthetic ligation product (bottom) *m/z* 666.0822, Δ *m/z* = 1.9 ppm.

2.12 Procedure for the reaction of synthetic isonitriles with AMI probe 3

Synthetic tyrosine-, tryptophan-, or valine-derived isonitriles (**6**, **11**, and **12**, 50 µM final concentration) were incubated with AMI probe **3** (50 µM final concentration) in a 1:1 mixture of freshly prepared ABC buffer (50 mM, pH 7.0) and organic solvent (MeOH or MeCN) overnight. The sample was then filtered and analyzed by HPLC-HRMS.

2.13 Reaction between AMI 3 and tryptophan- and valine isonitriles 11 and 12

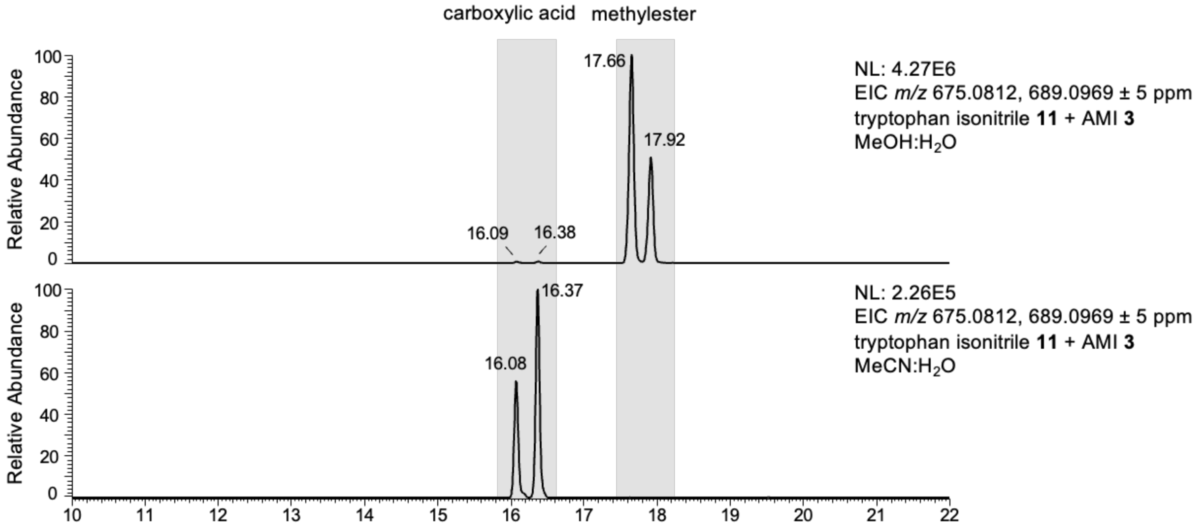

Figure S10: Extracted ion chromatograms (EICs) for *m/z* 675.0812 and 689.0969 ± 5 ppm, corresponding to the calculated [M+H] ions of the ligation products between AMI **3** and isonitrile **11**. Top chromatogram: Reaction performed with MeOH as organic co-solvent. Bottom chromatogram: Reaction performed with MeCN as organic co-solvent.

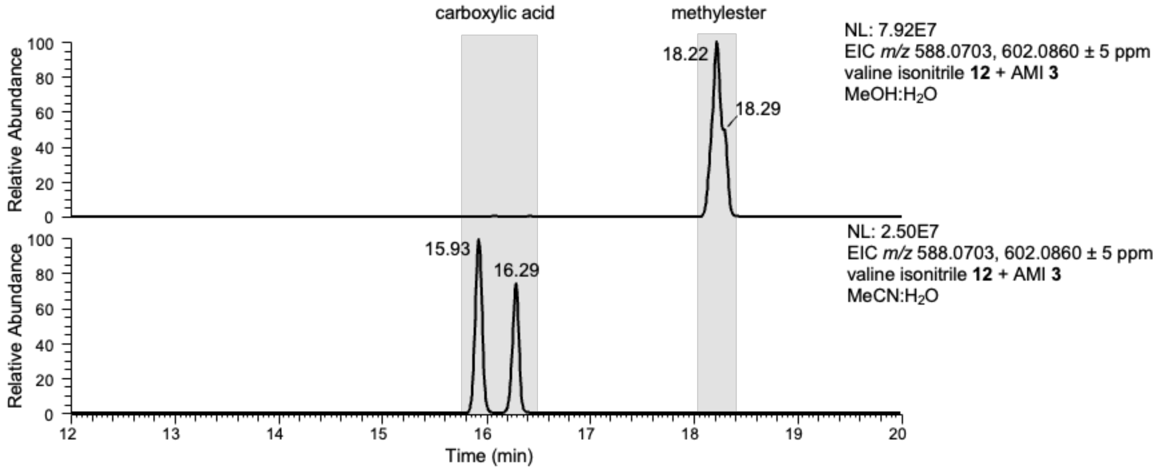

Figure S11: Extracted ion chromatograms (EICs) for *m/z* 588.0703 and 602.0860 ± 5 ppm, corresponding to the calculated $[M+H]^+$ ions of the ligation products between AMI **3** and isonitrile **12**. Top chromatogram: Reaction performed with MeOH as organic co-solvent. Bottom chromatogram: Reaction performed with MeCN as organic co-solvent. In the top chromatogram, signals corresponding to the calculated $[M+H]^+$ ions of the carboxylic acid were observed but are not visible in the chromatogram due to low intensities. The sample corresponding to the bottom chromatogram was quenched with 1 M HCl prior to the measurement.

2.14 Antibacterial assays

Table S1: Strains used for antibacterial assays

Species	Strain	Gram
Escherichia coli	DH5α	-
Bacillus subtilis	NCIB 3610[35]	+
Staphylococcus aureus	DSMZ 4910	+
Salmonella enterica	SB300 (serovar Typhimurium)	-
Stenotrophomonas maltophilia	Wild isolate	-

2.14.1 MIC_{50} assays

Single colonies of each bacterial strain were picked into Mueller–Hinton II Broth (MHB; BBL™, 10 mL) and cultivated at 30 °C (or 37 °C for *E. coli*) and 180 rpm overnight. On the following day, the overnight culture was diluted in fresh MHB to an optical density at 600 nm (OD_{600}) of 0.001. The compounds to be tested for antibacterial activity were serially diluted to final concentrations ranging from 16 mM to 15.62 µM. For the assay, the bacterial suspension (OD_{600} = 0.001, 50 µL) and the antibacterial compound (50 µL) were added to each well of a sterile 96-well microplate, resulting in a final volume of 100 µL per well. The filled plates were agitated for mixing purposes, briefly centrifuged to remove bubbles, and incubated statically at 37 °C for 24 h. After incubation, OD_{600} values were measured using a microplate reader. Percent survival was calculated by normalization to the OD_{600} of the no-compound control group.

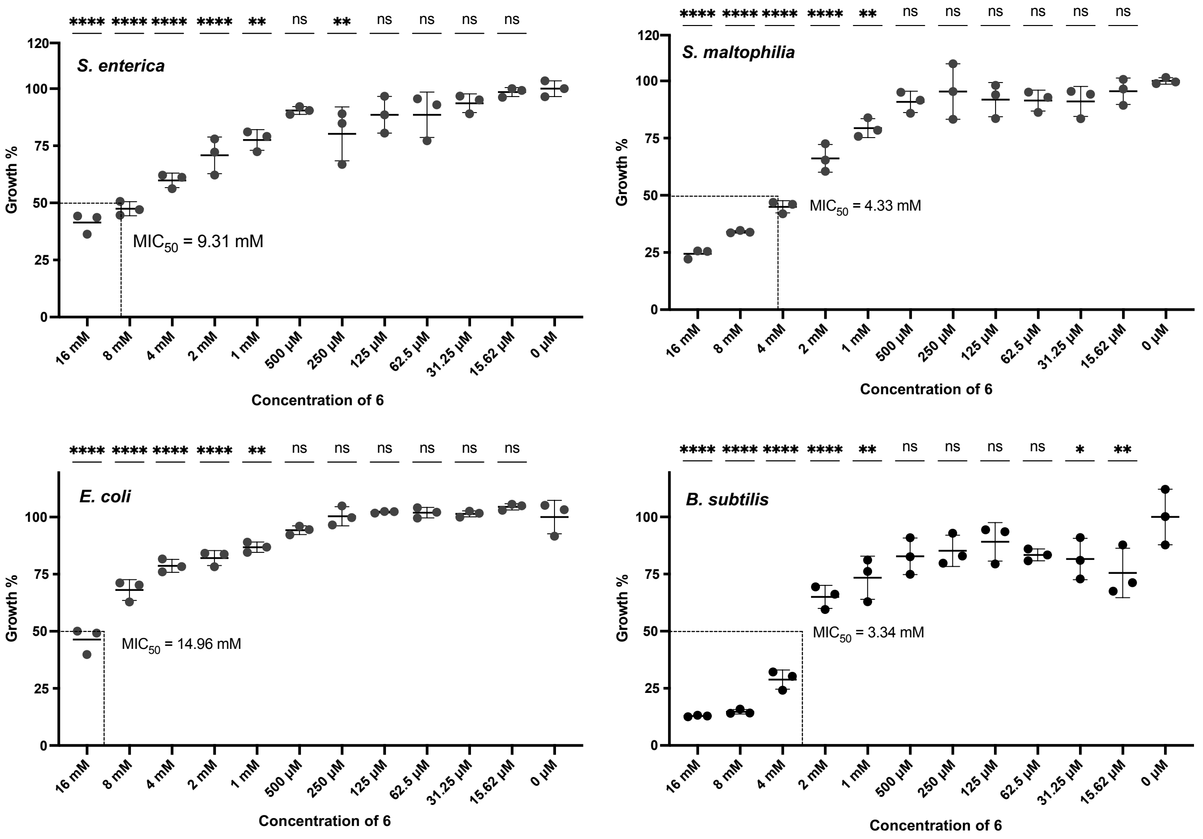

Figure S12: Growth inhibition of *S. enterica* SB300 (serovar typhimurium), *S. maltophilia* (wild isolate), *E. coli* (DH5α), *and B. subtilis* (NCIB 3610) by isonitrile **6**. MIC_{50} values were determined after 24 h. The error bars represent the mean ± SD from three independent biological replicates. A one-way ANOVA with Dunnett's multiple comparisons test was conducted to determine whether bacterial growth differed significantly across the tested concentration range compared to growth in medium alone. Statistical significance was set at $p < 0.05$. **** $p < 0.0001$; ns, not significant.

2.14.2 Disc assays

Single colonies of the tested bacterial strains were inoculated into LB medium (10 mL) and cultivated at 28 °C and 160 rpm overnight. 1 mL of each culture was then transferred into fresh LB medium (50 mL) and incubated at 30 °C until reaching the mid-logarithmic phase (OD_{600} = 0.6–0.8). A total of 200 µL of bacterial culture was spread evenly onto LB agar plates. Sterile filter discs were loaded with aqueous solutions containing defined amounts of the tested compounds (ampicillin, streptomycin, or isonitrile **6**) and air-dried completely in a sterile bench. The discs were then placed on the inoculated plates. All plates were incubated at 28 °C for 16 h, and antibacterial effects were evaluated visually based on inhibition zones.

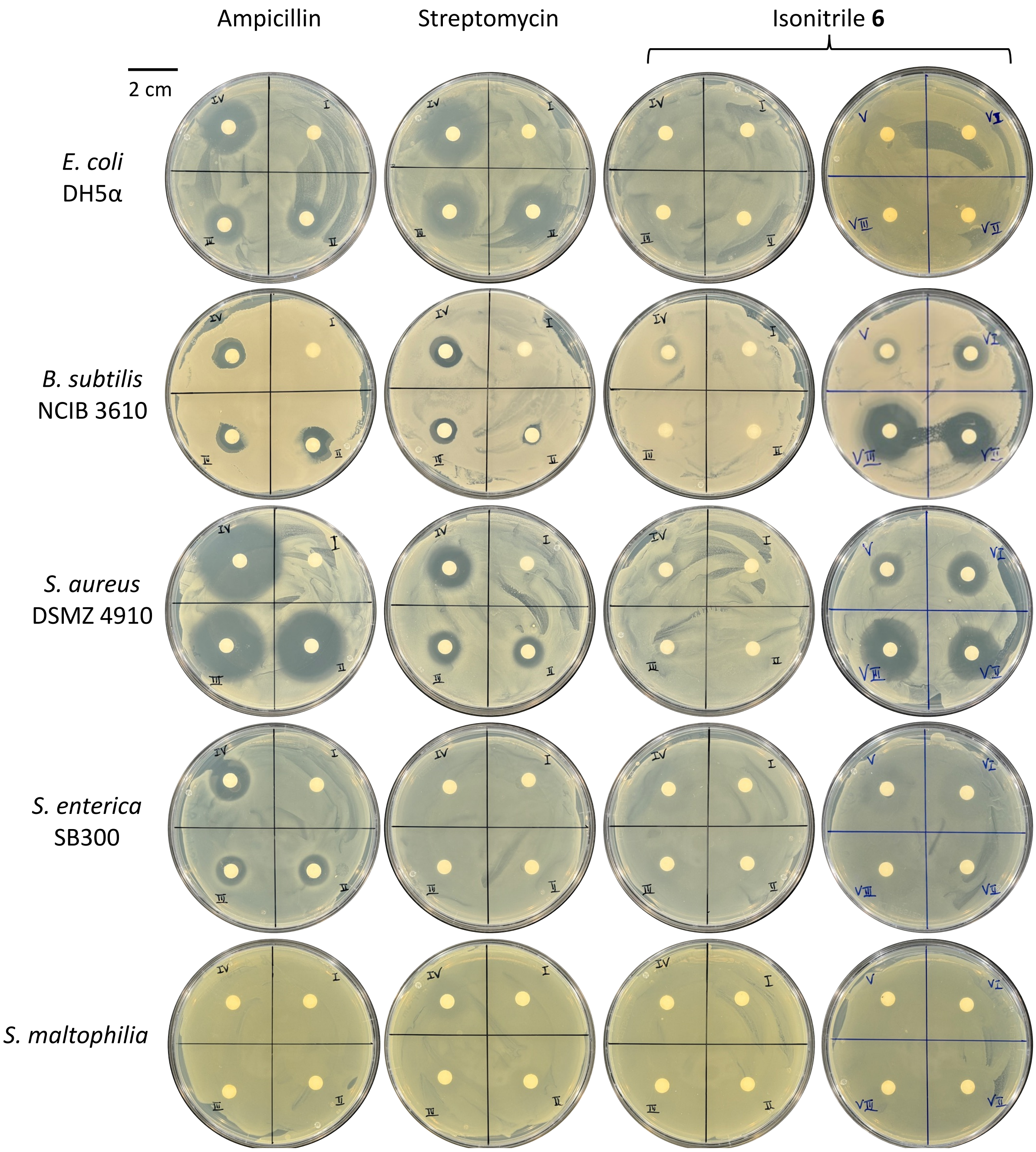

Figure S13: Growth inhibition of *E. coli*, *B. subtilis*, *S. aureus*, *S. enterica*, and *S. maltophilia* after treatment with ampicillin, streptomycin, or isonitrile **6**. Amount of compound per disc: I: 0 µg, II: 10 µg, III: 20 µg, IV: 50 µg, V: 100 µg, VI: 200 µg, VII: 400 µg, VIII: 800 µg.

2.15 UV-Vis absorption spectrum of probe 3

The absorption spectrum of **3** was recorded in water:MeCN + 0.1% formic acid (1:1), a typical solvent mixture for LC-MS. The probe was dissolved at a concentration of 40 µM and placed into a quartz cuvette with a path length of 1 cm to record the absorption spectrum.

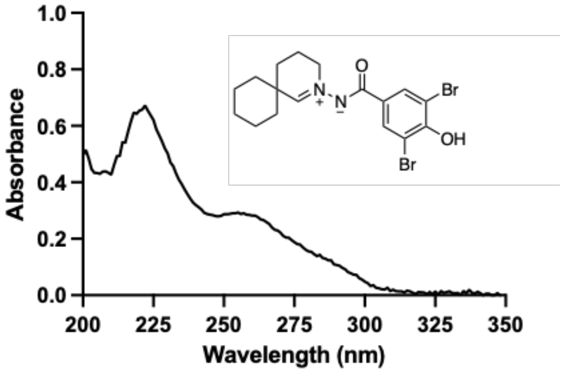

Figure S14: UV-Vis absorption spectrum of probe **3** in water:MeCN + 0.1% formic acid (1:1).

3 Synthetic procedures

Chlorooxime probe **3**, tryptophan isonitrile **11**, and valine isonitrile **12** were synthesized according to literature procedures.[20,32,36]

3.1 Synthesis of probe 3

Probe **3** was synthesized in two steps from 1-(3-bromopropyl)cyclohexane-1-carbaldehyde, which was prepared in two steps according to a literature procedure.[37]

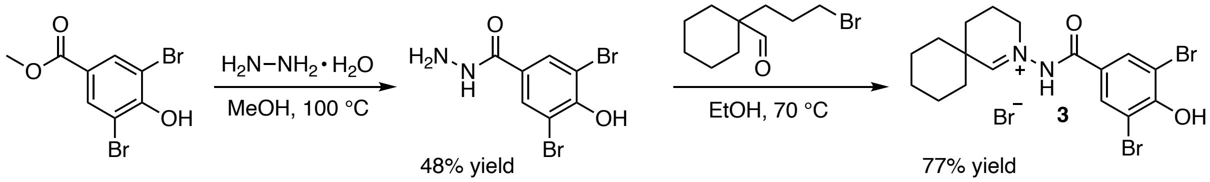

Scheme S1: Synthesis of probe **3**.

3,5-dibromo-4-hydroxybenzohydrazide

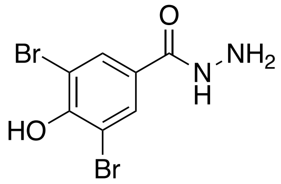

Methyl 3,4-dibromo-4-hydroxybenzoate (1 eq, 2.83 mmol, 877 mg) was dissolved in MeOH (7 mL) and hydrazine hydrate (80% solution in water, 7 mL) in a pressure vial. The vial was capped and heated to 100 °C for 6 h. The solution was cooled to room temperature and concentrated *in vacuo*. The white residue was dissolved in water:MeCN (1:1), frozen, and lyophilized. To remove residual hydrazine, the crude mixture was suspended in cold water (5 mL) and centrifuged. The supernatant was discarded, the pellet dissolved in water:MeCN (1:1), frozen, and lyophilized to yield the product as a white solid (420 mg, 1.36 mmol, 48%).

^{1}H NMR (400 MHz, CD_3OD) δ 7.93 (s, 2H). **^{13}C NMR** (126 MHz, DMSO-*d_6*) δ 163.5, 155.0, 131.0, 124.7, 111.9. **HRMS** (ESI): *m/z* calcd. for $C_7H_7Br_2N_2O_2$ $[M + H]^+$: 308.8869; found: 308.8865

2-(3,5-dibromo-4-hydroxybenzamido)-2-azaspiro[5.5]undec-1-en-2-ium bromide (3)

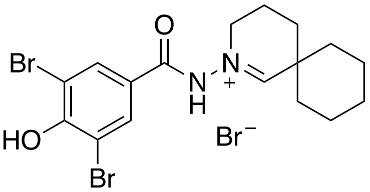

3,5-dibromo-4-hydroxybenzohydrazide (1 eq, 216 µmol, 67 mg) and 1-(3-bromopropyl)cyclohexane-1-carbaldehyde (1.3 eq, 281 µmol, 66 mg) were suspended in EtOH (1 mL) and heated for 2 h at 70 °C. The resulting yellow solution was cooled to room temperature and poured into a falcon tube containing cold diethyl ether upon which the product crashed out. The falcon tube was centrifuged and the supernatant decanted. The pellet was washed with diethyl ether once and then dried *in vacuo*, which yielded the product as a light brown solid (77 mg, 167 µmol, 77%).

^{1}H NMR (600 MHz, CD_3OD) δ 8.93 (brs, 1H), 8.09 (s, 2H), 4.02 (brs, 2H), 2.13 (brs, 2H), 2.01 – 1.23 (m, 14H). **^{13}C NMR** (151 MHz, CD_3OD) δ 187.9, 164.2, 157.2, 133.6, 124.6, 112.1, 56.8, 42.5, 34.5, 27.3, 26.1, 21.2, 19.5. **HRMS** (ESI): *m/z* calcd. for $C_{17}H_{21}Br_2N_2O_2$ $[M - Br]^+$: 442.9964; found: 442.9958.

3.2 Synthesis of isonitrile 6

Isonitrile **6** was synthesized in two steps from commercially available H-Tyr-OMe. Spectroscopic data were in agreement with those reported.[38]

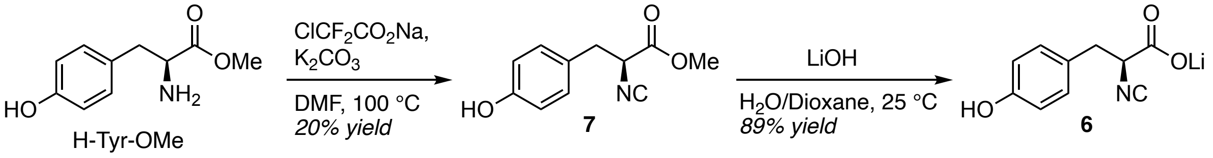

Scheme S2: Synthesis of isonitrile **6**.

Methyl (*S*)-3-(4-hydroxyphenyl)-2-isocyanopropanoate (7)

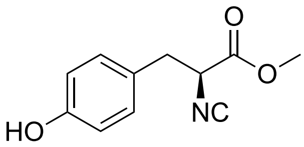

H-Tyr-OMe (1 eq, 1.6 mmol, 312 mg), potassium carbonate (2 eq, 3.2 mmol, 442 mg) and sodium chlorodifluoroacetate (2 eq, 3.2 mmol, 487 mg) were placed in a microwave vial. Dry DMF (15 mL) was added, and argon was bubbled through the reaction mixture. The suspension was heated in a microwave reactor (7 h, 100 °C) and the crude reaction mixture was diluted with EtOAc and then washed with 5% aq. LiCl (1x) and finally brine (1x). The organic phase was dried over magnesium sulfate, filtered, and purified by silica flash column chromatography (EtOAc in hexanes, 20% to 50%). The target compound was isolated as a yellow oil (66 mg, 322 µmol, 20%).

^{1}H NMR (500 MHz, DMSO-d_6) δ 9.36 (s, 1H), 7.03 (d, *J* = 8.6 Hz, 2H), 6.71 (d, *J* = 8.6 Hz, 2H), 5.02 (dd, *J* = 7.8, 4.9 Hz, 1H), 3.73 (s, 3H), 3.17 – 2.88 (m, 2H). **^{13}C NMR** (126 MHz, DMSO-d_6) δ 166.9, 159.2, 156.7, 130.4, 125.0, 115.2, 57.8, 53.1, 36.9. **HRMS** (ESI): *m/z* calcd. for $C_{11}H_{12}NO_3$ $[M + H]^+$: 206.0812; found: 206.0817

Lithium (S)-3-(4-hydroxyphenyl)-2-isocyanopropanoate (6)

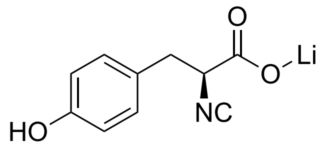

Methyl (*S*)-3-(4-hydroxyphenyl)-2-isocyanopropanoate **7** (1 eq, 297 µmol, 61 mg) was dissolved in dioxane (500 µL) and water (54 µL). LiOH monohydrate (1.5 eq, 446 µL of a 1 M aqueous solution) was added and the solution was stirred at room temperature for 30 min. The solution was then diluted with water:MeCN (1:1), frozen, and lyophilized. The product was obtained as an off-white solid (56 mg) that contained 0.5 eq (149 µmol, 4 mg) residual LiOH, resulting in a yield of 89%.

^{1}H NMR (500 MHz, D_2O) δ 7.13 (d, *J* = 8.5 Hz, 2H), 6.70 (d, *J* = 8.6 Hz, 2H), 4.52 – 4.29 (m, 1H), 3.23 – 2.88 (m, 2H). **^{13}C NMR** (126 MHz, D_2O) δ 173.2, 162.4, 153.1 (t, *J* = 6.8 Hz, isocyanide carbon), 130.7, 123.6, 117.77, 61.3 (t, 6.9 Hz, α-carbon), 37.9. **HRMS** (ESI): *m/z* calcd. for $C_{10}H_8NO_3$ $[M - H]^-$: 190.051; found: 190.0509

3.3 Synthesis of ligation product 10b

Methyl ((*R*/*S*)-2-(3,5-dibromo-4-hydroxybenzamido)-2-azaspiro[5.5]undecane-1-carbonyl)-*L*-tyrosinate (10b)

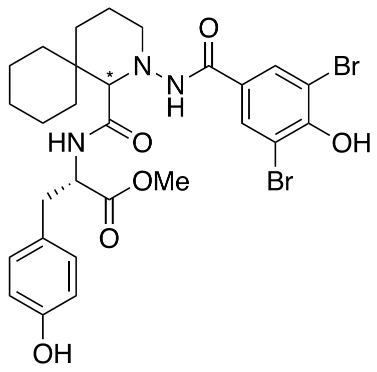

AMI probe **3** (1 eq, 31 µmol, 16.3 mg) was suspended in MeCN (1 mL) and sodium phosphate buffer (50 mM, pH 7.0, 1 mL). Isonitrile **7** (1.4 eq, 43.5 µmol, 8.9 mg) dissolved in MeCN (1 mL) was added. After stirring at room temperature for 4 h, aq. HCl (1 M, 0.5 mL) was added to the light yellow solution and after stirring for another 30 min the solution was frozen and lyophilized. The crude mixture was purified by reverse-phase MPLC (MeCN in water + 0.1% HCl, 20% to 80%). The product was obtained as white powder (2.7 mg, 4.1 µmol, 13%).

NMR spectroscopy showed a ca. 9:1 mixture of two diastereomers.
^{1}H NMR (600 MHz, CD_3OD + 5% aq. HCl (1 M), measured with suppression of the water signal) δ 8.00 (s, 2H), 7.00 (d, *J* = 8.5 Hz, 2H), 6.67 (d, *J* = 8.6 Hz, 2H), 4.58 (s, 1H), 3.60 (s, 3H), 3.55 (br, 1H), 3.08 – 2.99 (m, 1H), 2.90 (br, 1H), 2.79 (dd, *J* = 14.4, 9.5 Hz, 1H), 2.04 (br, *J* = 12.1 Hz, 1H), 1.77 (br, 2H), 1.56 – 1.04 (m, 14H). **^{13}C NMR** (151 MHz, CD_3OD + 5% aq. HCl (1 M), measured with suppression of the water signal) δ 173.5, 165.3, 157.3, 155.9, 133.2, 131.0, 128.6, 127.0, 116.3, 111.9, 55.3, 52.8, 46.1, 38.2, 37.1, 35.2, 27.2, 24.3, 22.1, 22.0, 20.8. **HRMS** (ESI): *m/z* calcd. for $C_{28}H_{34}Br_2N_3O_6$ $[M + H]^+$: 666.0809; found: 666.0805

4 X-ray crystallographic data

Crystal structure of methyl ((*R*)-2-(3,5-dibromo-4-hydroxybenzamido)-2-azaspiro[5.5]undecane-1-carbonyl)-L-tyrosinate

Single crystals of **10b** were obtained from the NMR solution (CD_3OD + 5% HCl (1 M)) by slow solvent evaporation. A suitable crystal was selected and measured on a XtaLAB Synergy R, HyPix-Arc 150 diffractometer. The crystal was kept at 100.0(1) K during data collection. Using Olex2,[39] the structure was solved with the SHELXT[40] structure solution program using Intrinsic Phasing and refined with the SHELXL[41] refinement package using Least Squares minimization.

Crystal size/mm^3: 0.15 × 0.104 × 0.059.

Crystal Data for $C_{28}H_{33}N_3O_6Br_2$ (*M* = 667.39 g/mol): triclinic, space group P-1, *a* = 10.50500(10) Å, *b* = 12.48980(10) Å, *c* = 12.85210(10) Å, *V* = 1413.16(2) Å^3, Z = 2, T = 100.0(1) K, μ(Cu Kα) = 1.54184 mm^{-1}, *Dcalc* = 1.568 g/cm^3, 54142 reflections measured (8.18° ≤ 2Θ ≤ 149.022°), 5550 unique (R_{int} = 0.0222, R_{sigma} = 0.0106) which were used in all calculations. The final R_1 was 0.0246 (I > 2σ(I)) and wR_2 was 0.0610 (all data).

The crystal structure is deposited in the Cambridge Crystallographic Data Centre (CCDC Code: 2499029).

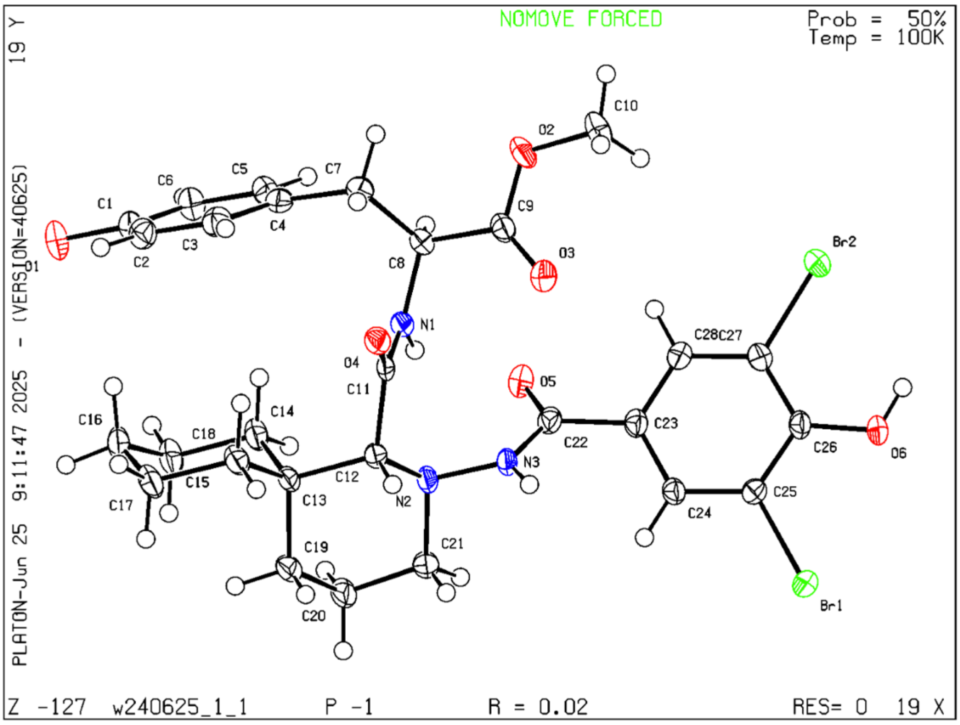

Figure S15: Crystal structure of methyl ((*R*)-2-(3,5-dibromo-4-hydroxybenzamido)-2-azaspiro[5.5]undecane-1-carbonyl)-*L*-tyrosinate with thermal ellipsoids at 50% probability.

5 PSI-BLAST query

Table S2: Homolog candidates to L-tyrosine isonitrile synthase (IsnA, WP_010845414.1) from *Xenorhabdus nematophila*.

Species	Gene Name	NCBI Accession	Annotation	Length (aa)	Homology (%)	E-value
Phycomyces blakesleeanus NRRL 1555	PHYBLDRAFT_71450	XP_018292322.1	Hypothetical protein	428	33.07	2E-30
Aspergillus nidulans FGSC A4	ANIA_02606	XP_660210.1	Hypothetical protein	661	30.87	3E-30
Aspergillus nidulans FGSC A4	ANIA_02705	XP_660309.1	Protein DitA	664	68.00	5E-28

6 References cited only Supporting Information

[35] A. Tayyrov, C. E. Stanley, S. Azevedo and M. Künzler, *BMC Genom.*, **2019**, *20*, 243.

[36] W.-C. Chang, D. Sanyal, J.-L. Huang, K. Ittiamornkul, Q. Zhu and X. Liu, *Org. Lett.*, **2017**, *19*, 1208-1211.

[37] T. Hashimoto, Y. Maeda, M. Omote, H. Nakatsu and K. Maruoka, *J. Am. Chem. Soc.*, **2010**, *132*, 4076-4077.

[38] C.-P. Yu, Y. Tang, L. Cha, S. Milikisiyants, T. I. Smirnova, A. I. Smirnov, Y. Guo and W.-c. Chang, *J. Am. Chem. Soc.*, **2018**, *140*, 15190-15193.

[39] O. V. Dolomanov, L. J. Bourhis, R. J. Gildea, J. A. Howard and H. Puschmann, *Appl. Crystallogr.*, **2009**, *42*, 339-341.

[40] G. M. Sheldrick, *Found. Crystallogr.*, **2015**, *71*, 3-8.

[41] G. M. Sheldrick, *Acta Crystallogr. C Struct. Chem.*, **2015**, *71*, 3-8.

7 ^{1}H and ^{13}C NMR spectra

^{1}H NMR (400 MHz, CD_3OD) of 3,5-dibromo-4-hydroxybenzohydrazide

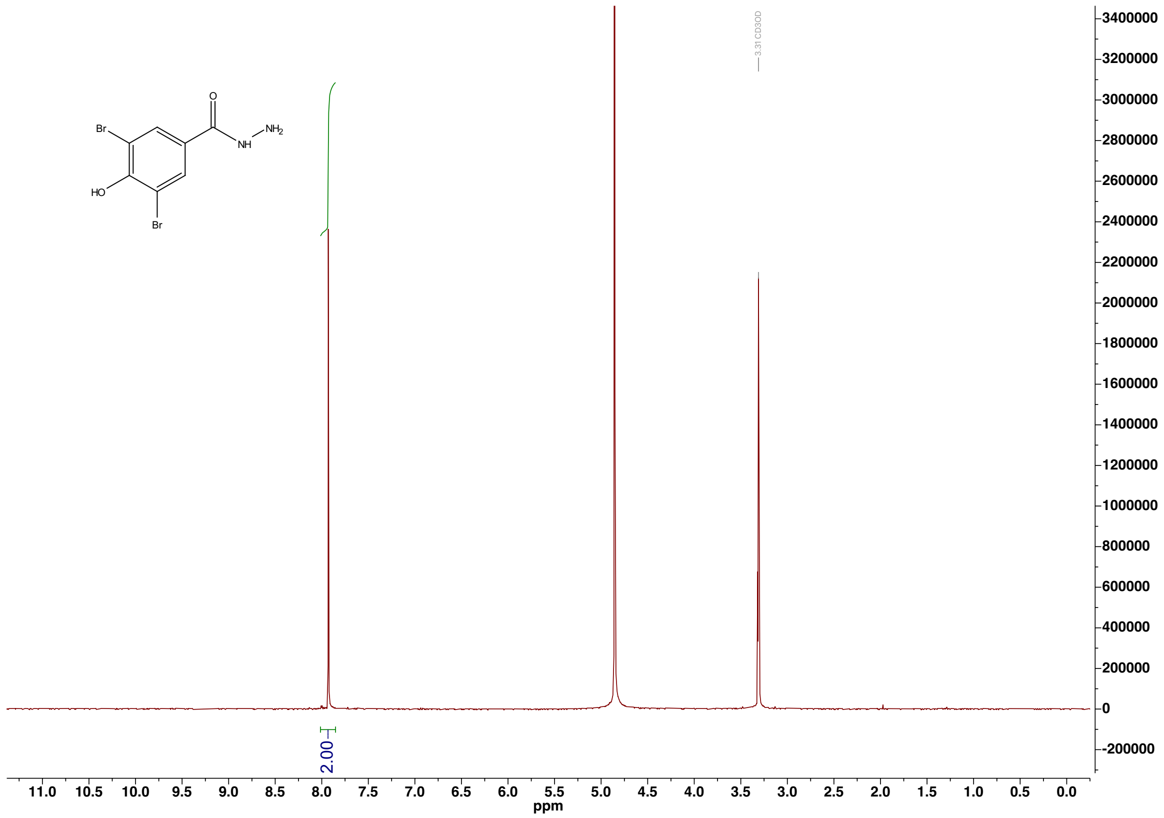

^{13}C NMR (126 MHz, DMSO-d_6) of 3,5-dibromo-4-hydroxybenzohydrazide

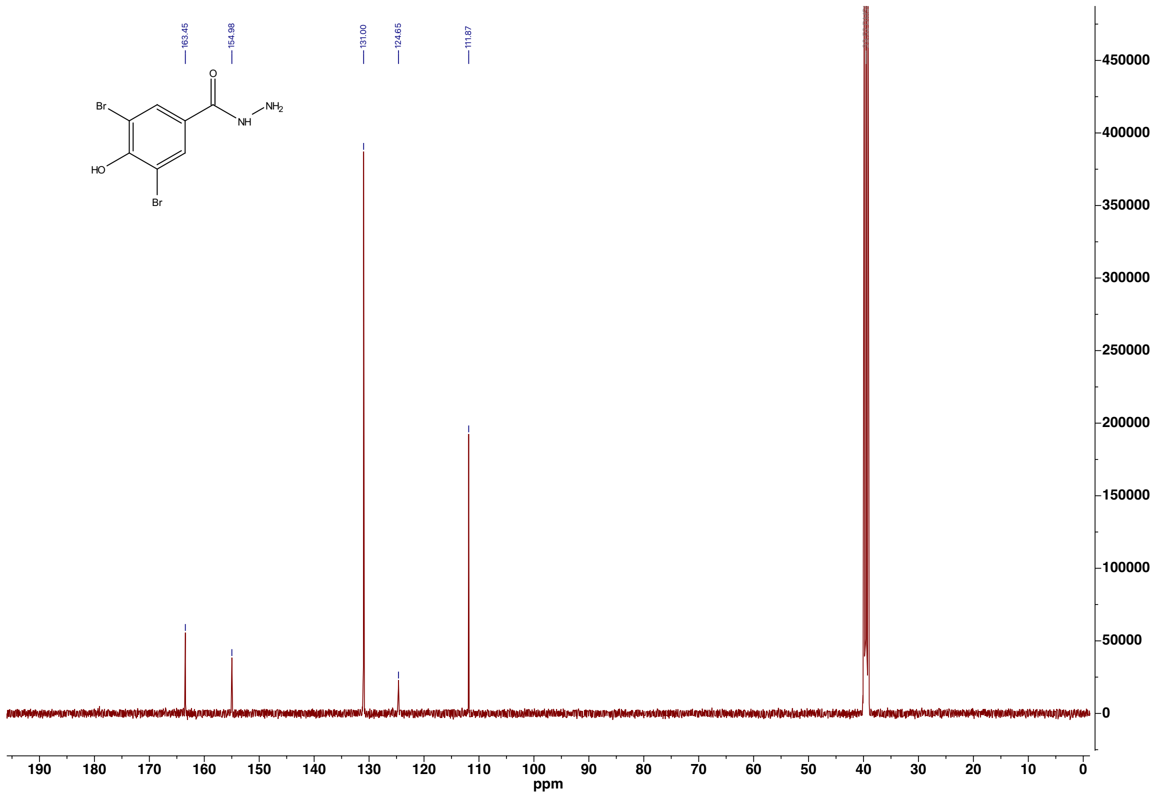

^{1}H NMR (600 MHz, CD_3OD) of **3**

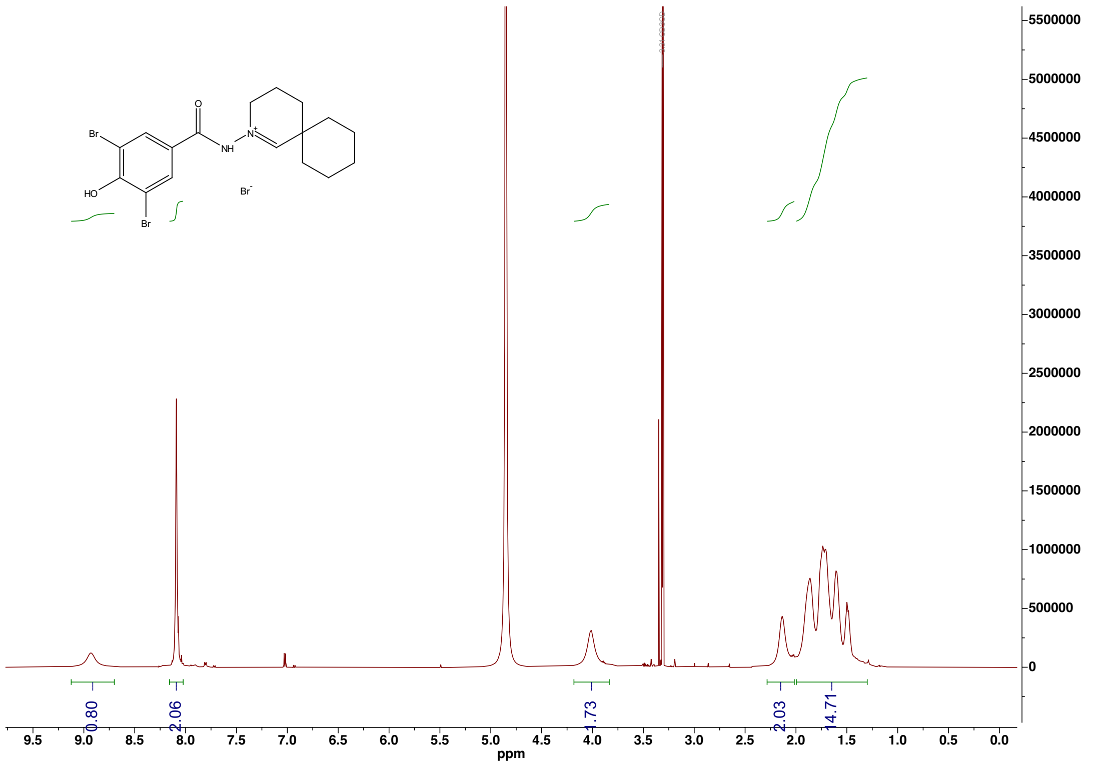

^{13}C NMR (151 MHz, CD_3OD) of **3**

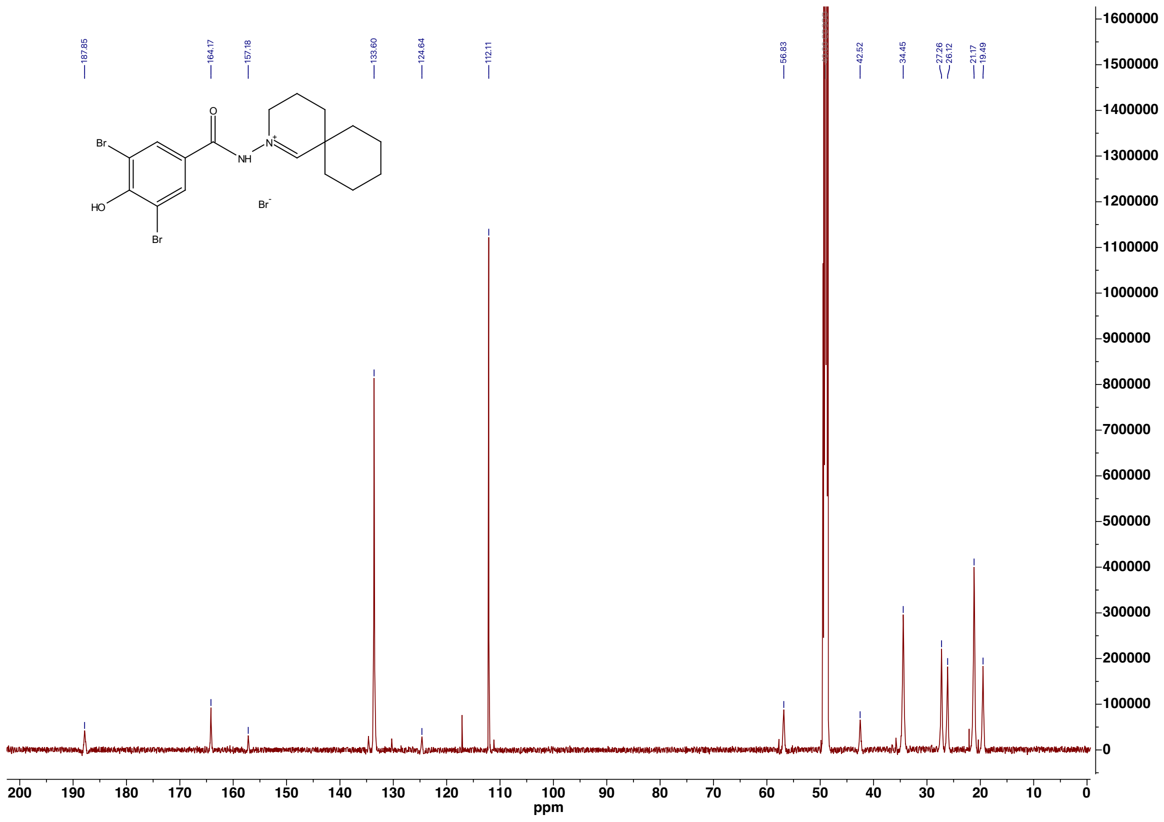

^{1}H NMR (500 MHz, DMSO-d_6) of **7**

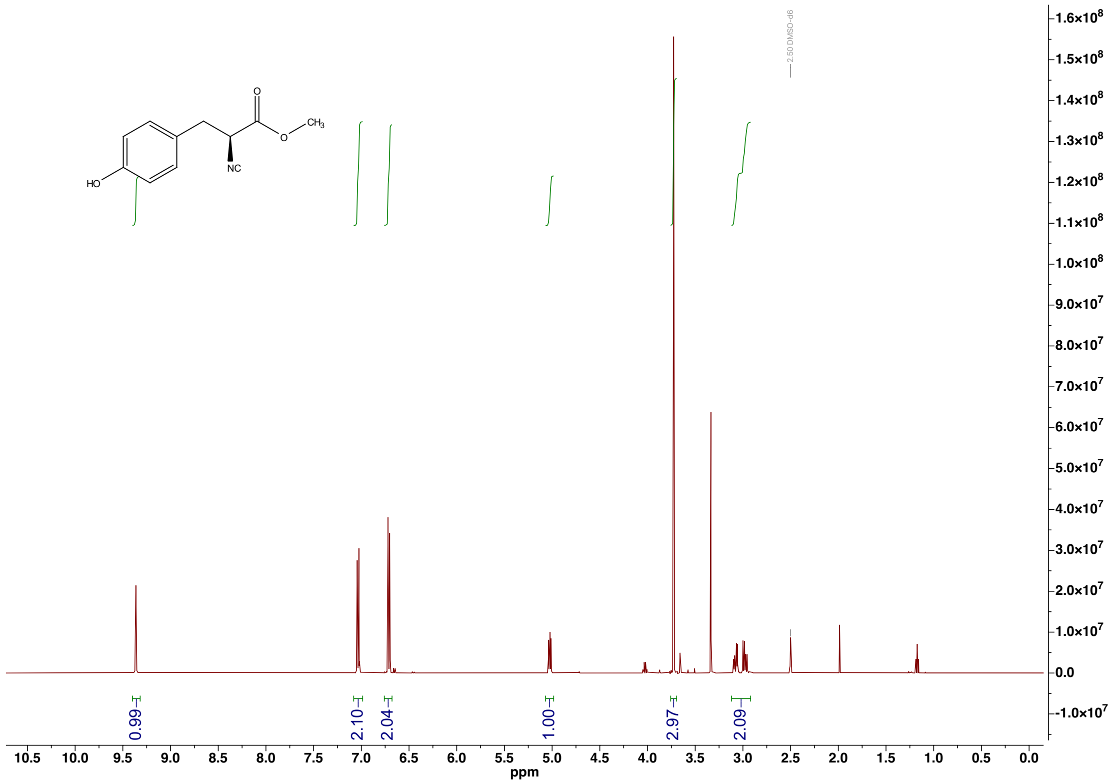

^{13}C NMR (126 MHz, DMSO-d_6) of **7**

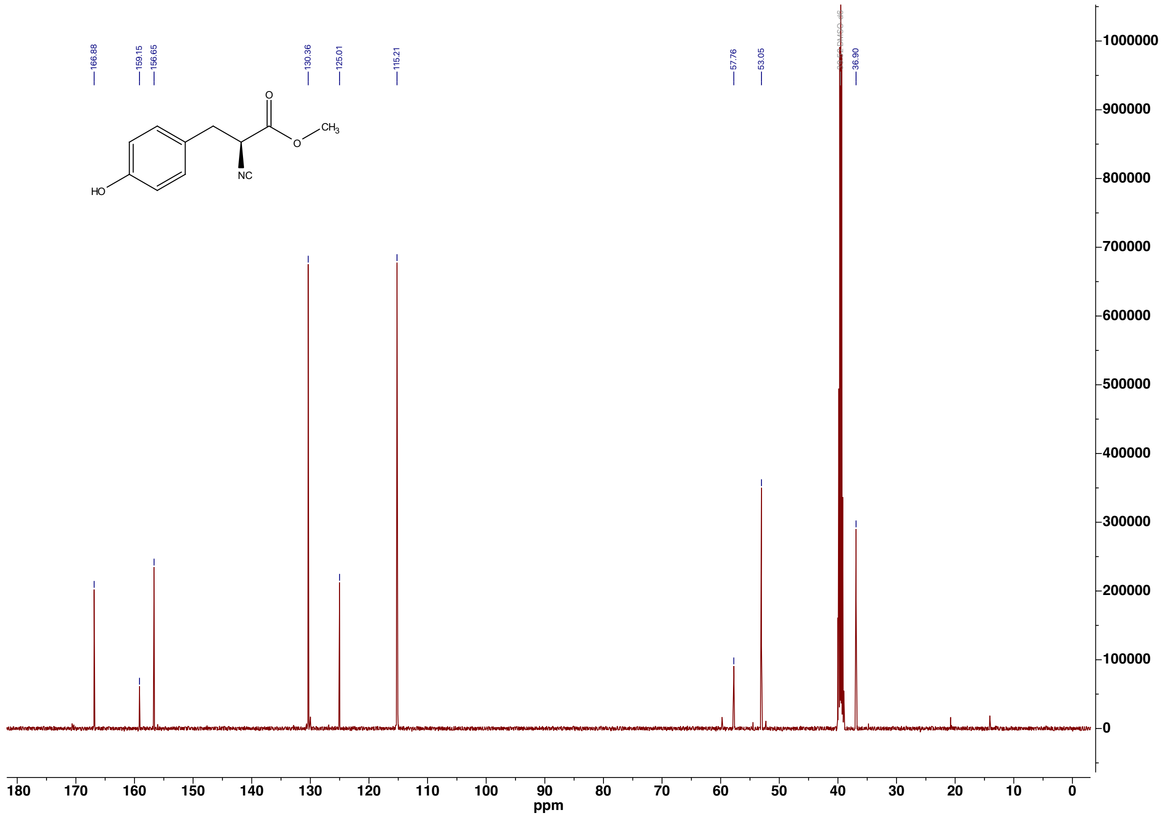

^{1}H NMR (500 MHz, D_2O) of **6**

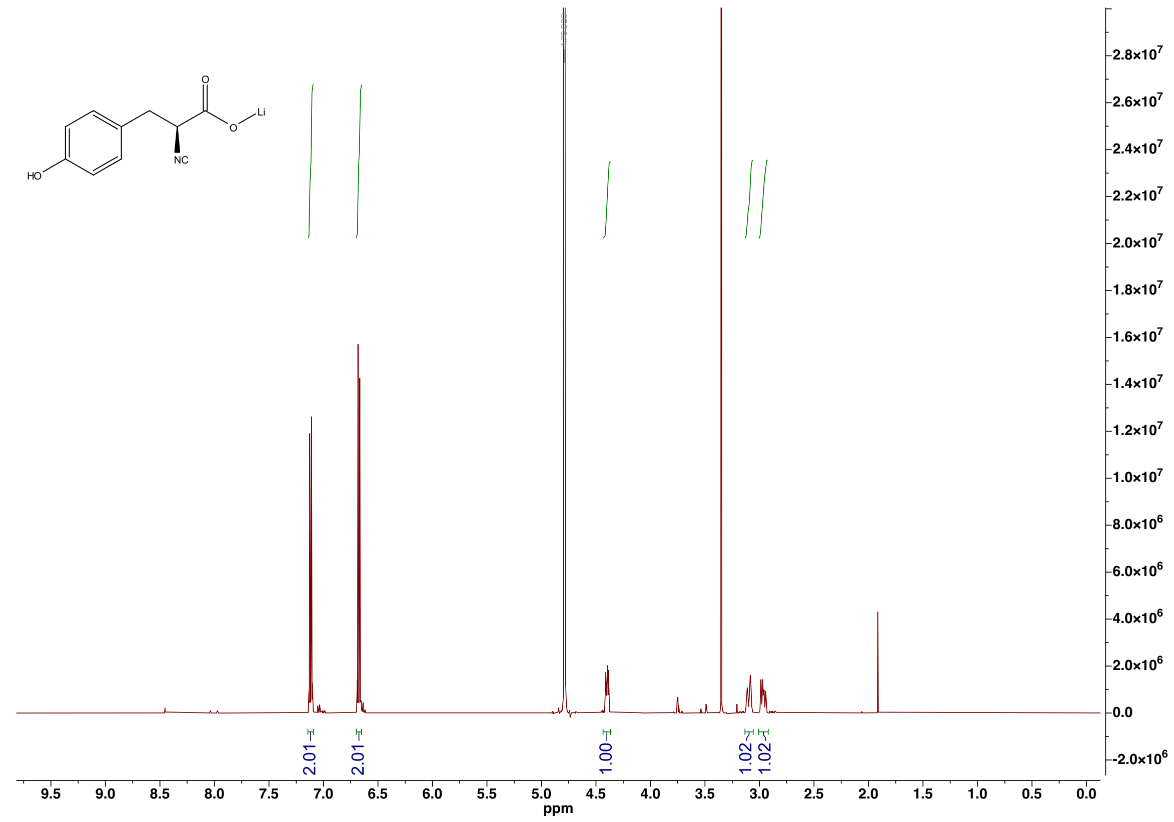

^{13}C NMR (126 MHz, D_2O) of **6**

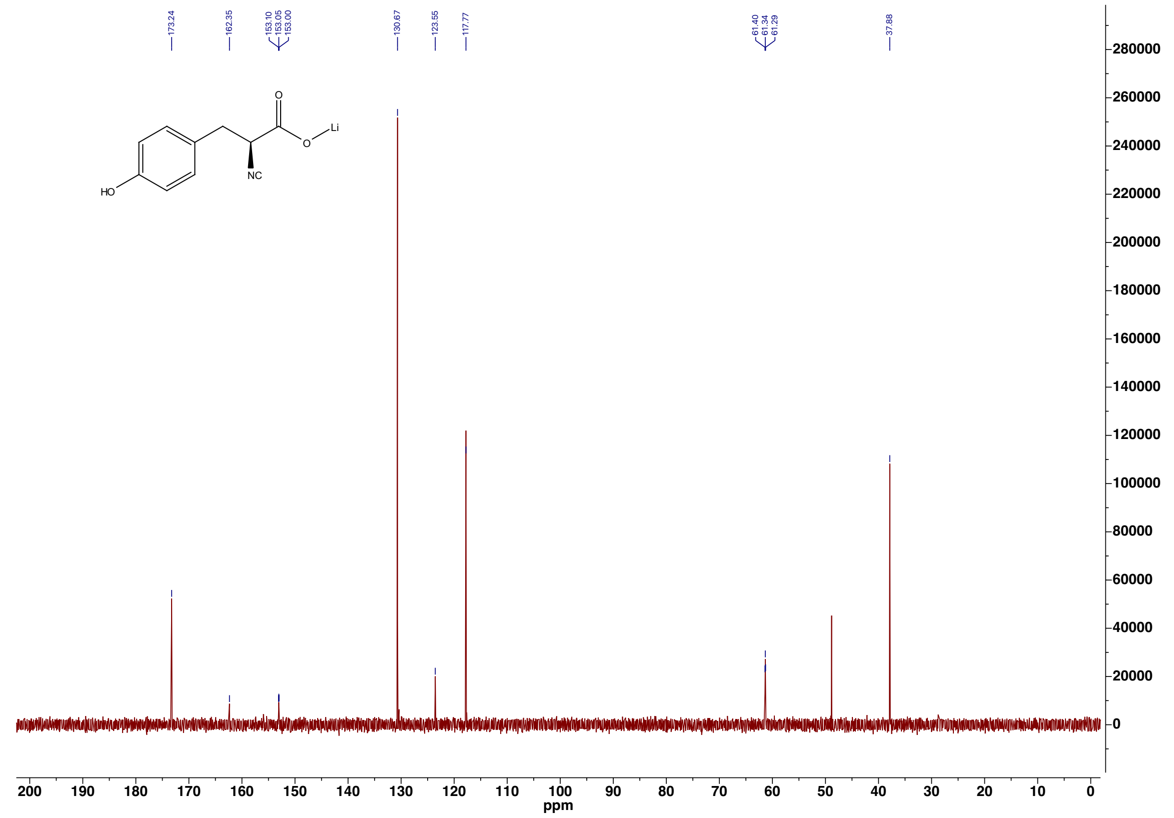

^{1}H NMR (600 MHz, CD_3OD + 5% aq. HCl (1 M)) of **10b**

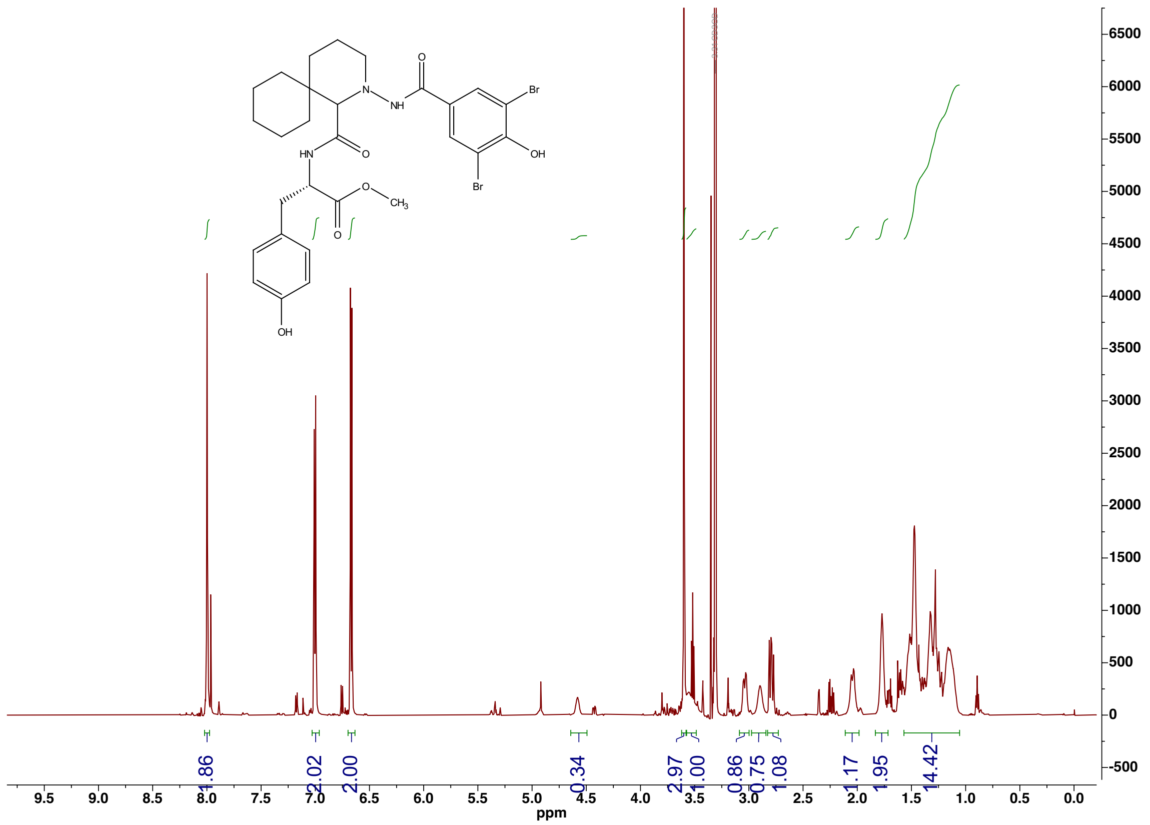

^{13}C NMR (151 MHz, CD_3OD + 5% HCl (1 M)) of **10b**

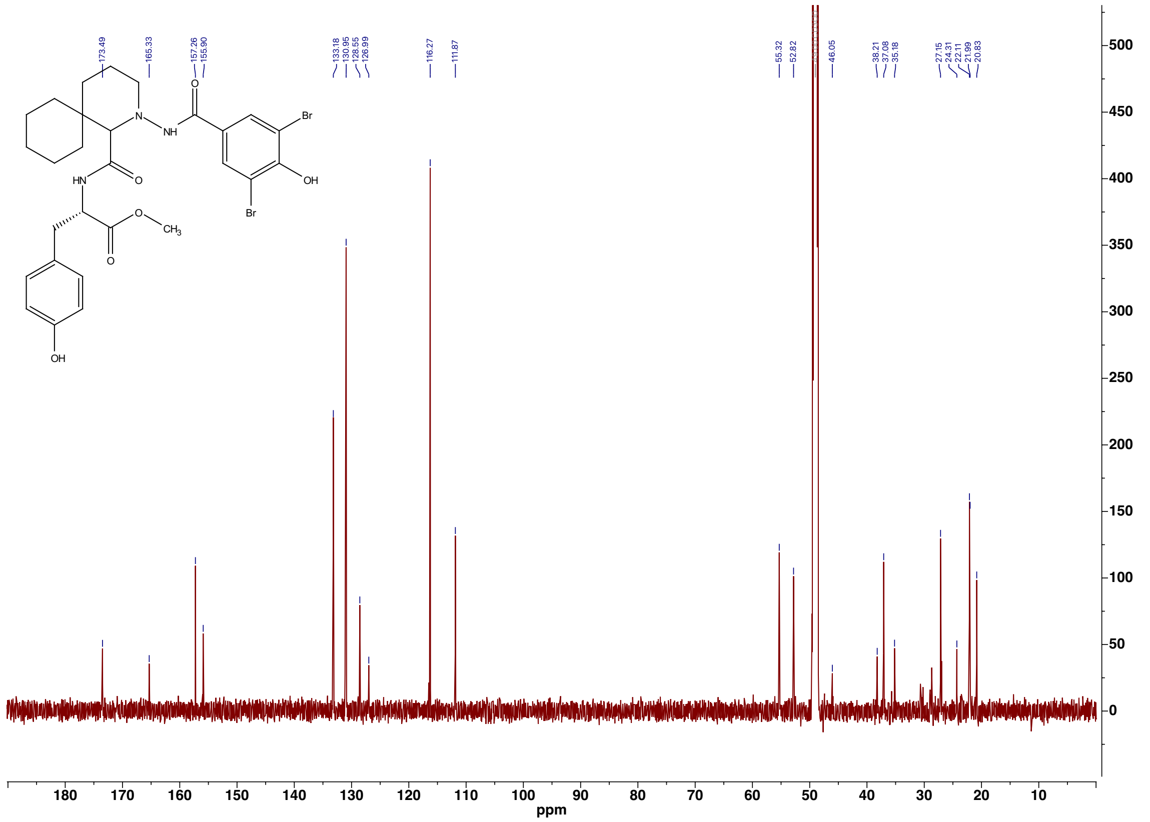

Supplement: Supplementary file 2 — Supporting File 1: chem70671‐sup‐0002‐Data.zip. [file CHEM-32-e03642-s001.pdf]
